# Stroke and myocardial infarction induce neutrophil extracellular trap release disrupting lymphoid organ structure and immunoglobulin secretion

**DOI:** 10.1038/s44161-024-00462-8

**Published:** 2024-04-23

**Authors:** Ali A. Tuz, Susmita Ghosh, Laura Karsch, Dimitris Ttoouli, Sai P. Sata, Özgür Ulusoy, Andreas Kraus, Nils Hoerenbaum, Jan-Niklas Wolf, Sabrina Lohmann, Franziska Zwirnlein, Viola Kaygusuz, Vivian Lakovic, Hannah-Lea Tummes, Alexander Beer, Markus Gallert, Stephanie Thiebes, Altea Qefalia, Zülal Cibir, Medina Antler, Sebastian Korste, Elias Haj Yehia, Lars Michel, Tienush Rassaf, Britta Kaltwasser, Hossam Abdelrahman, Ayan Mohamud Yusuf, Chen Wang, Dongpei Yin, Lars Haeusler, Smiths Lueong, Mathis Richter, Daniel R. Engel, Martin Stenzel, Oliver Soehnlein, Benedikt Frank, Mialitiana Solo-Nomenjanahary, Benoît Ho-Tin-Noé, Jens T. Siveke, Matthias Totzeck, Daniel Hoffmann, Anika Grüneboom, Nina Hagemann, Anja Hasenberg, Jean-Philippe Desilles, Mikael Mazighi, Albert Sickmann, Jianxu Chen, Dirk M. Hermann, Matthias Gunzer, Vikramjeet Singh

**Affiliations:** 1 Institute for Experimental Immunology and Imaging, University Hospital, University of Duisburg-Essen, Essen, Germany; 2 Leibniz-Institut für Analytische Wissenschaften - ISAS-e.V., Dortmund, Germany; 3 Bioinformatics and Computational Biophysics, Faculty of Biology and Centre for Medical Biotechnology (ZMB), University of Duisburg-Essen, Essen, Germany; 4 Department of Immunodynamics, Institute of Experimental Immunology and Imaging, University Hospital Essen, Essen, Germany; 5 Department of Cardiology and Vascular Medicine, West German Heart and Vascular Center, University Hospital, University of Duisburg-Essen, Essen, Germany; 6 Department of Neurology, University Hospital, University of Duisburg-Essen, Essen, Germany; 7 Division of Solid Tumor Translational Oncology, German Cancer Consortium (DKTK, partner site Essen), German Cancer Research Center (DKFZ), Heidelberg, Germany; 8 Institute for Experimental Pathology (ExPat), Center for Molecular Biology of Inflammation (ZMBE), Universität Münster, Münster, Germany; 9 Optimisation Thérapeutique en Neuropsychopharmacologie, Université Paris Cité, U1144 Institut National de la Santé et de la Recherche Médicale (INSERM), Paris, France; 10 Interventional Neuroradiology Department and Biological Resources Center, Rothschild Foundation Hospital, Paris, France; 11 Medizinisches Proteom-Center, Ruhr-Universität Bochum, Bochum, Germany; 12 Department of Chemistry, College of Physical Sciences, University of Aberdeen, Aberdeen, UK

**Keywords:** Plasma cells, Neutrophils

## Abstract

Post-injury dysfunction of humoral immunity accounts for infections and poor outcomes in cardiovascular diseases. Among immunoglobulins (Ig), IgA, the most abundant mucosal antibody, is produced by plasma B cells in intestinal Peyer’s patches (PP) and lamina propria. Here we show that patients with stroke and myocardial ischemia (MI) had strongly reduced IgA blood levels. This was phenocopied in experimental mouse models where decreased plasma and fecal IgA were accompanied by rapid loss of IgA-producing plasma cells in PP and lamina propria. Reduced plasma IgG was detectable in patients and experimental mice 3–10 d after injury. Stroke/MI triggered the release of neutrophil extracellular traps (NETs). Depletion of neutrophils, NET degradation or blockade of NET release inhibited the loss of IgA^+^ cells and circulating IgA in experimental stroke and MI and in patients with stroke. Our results unveil how tissue-injury-triggered systemic NET release disrupts physiological Ig secretion and how this can be inhibited in patients.

## Abstract (web summary)

Tuz et al. report that stroke and myocardial infarction induce the release of neutrophil extracellular traps (NETs), triggering the loss of B cells and a decrease in immunoglobulin A secretion, and that inhibition of NETs prevents the loss of immunoglobulin A in mice and in patients with stroke.

## Main

Ischemic stroke and myocardial infarction (MI) are life-threatening disorders with revascularization as the only therapeutic option^1^. Systemic immune dysfunction and infections are major complications in these disorders and are associated with poor clinical outcome^2^. Still, definite mechanisms and targeted therapies to rebalance post-injury immune defects do not exist. Tissue injury can trigger apoptosis of systemic lymphocytes, and the resulting immunosuppression can make patients vulnerable to microbial invasion, especially at mucosal barriers^3,4^. Intestinal B cells are the major source of immunoglobulin A (IgA)-producing plasma cells and play a fundamental role in the protection of mucosal barriers. Peyer’s patches (PP), which are central structures of gut-associated lymphoid tissues, contain substantial numbers of IgA-switched B cells that later migrate to the lamina propria (LP) and secrete IgA^5^. IgA is the most abundant secreted isotype at mucosal surfaces with a shorter half-life (<1 d) compared to IgG (3–7 d)^6^. Both IgA and IgG can bind to microbes and inhibit their invasion of epithelial cells^7^. Under homeostatic conditions, the generation of IgA-producing plasma cells occurs in the germinal centers (GCs) of PP and requires complex interactions among B cells, antigen-presenting cells and helper T cells^8^. At homeostasis, the constant exposure of PP to commensal microflora or food antigens also induces tolerance in immune cells, thus inhibiting unwanted inflammation and autoimmunity^9^. Patients with ischemic stroke have decreased humoral immunity and, thus, are at higher risk for bacterial infections^10^. Indeed, infections are a major reason for high mortality after stroke^11^. However, despite the important role of B cells in mucosal barrier defense and immune homeostasis, the effects of vascular disorders, such as stroke and MI, on intestinal B cells remain largely unexplored.

In the present study, we used experimental mouse models of ischemic stroke and MI and blood samples from patients with ischemic stroke and MI to identify the influence of these sterile inflammatory conditions on intestinal B and T cell numbers and circulating Ig levels. First, we detected strongly decreased circulating Ig levels in patients with stroke and MI and experimental animals. Using multiple molecular analyses and advanced imaging, we then found that the release of neutrophil extracellular traps (NETs) triggers a rapid and long-lasting loss of intestinal B and T cells, including Ig-producing plasma cells, which provides a rational explanation for the reduced amounts of circulating Ig. We also show that the degradation of NETs with DNase-I therapy inhibited the loss of circulating IgA in experimental mouse models and in patients with stroke.

## Ischemic stroke and MI reduce the volumes of PP and the levels of systemic Ig

**Fig. 1:**
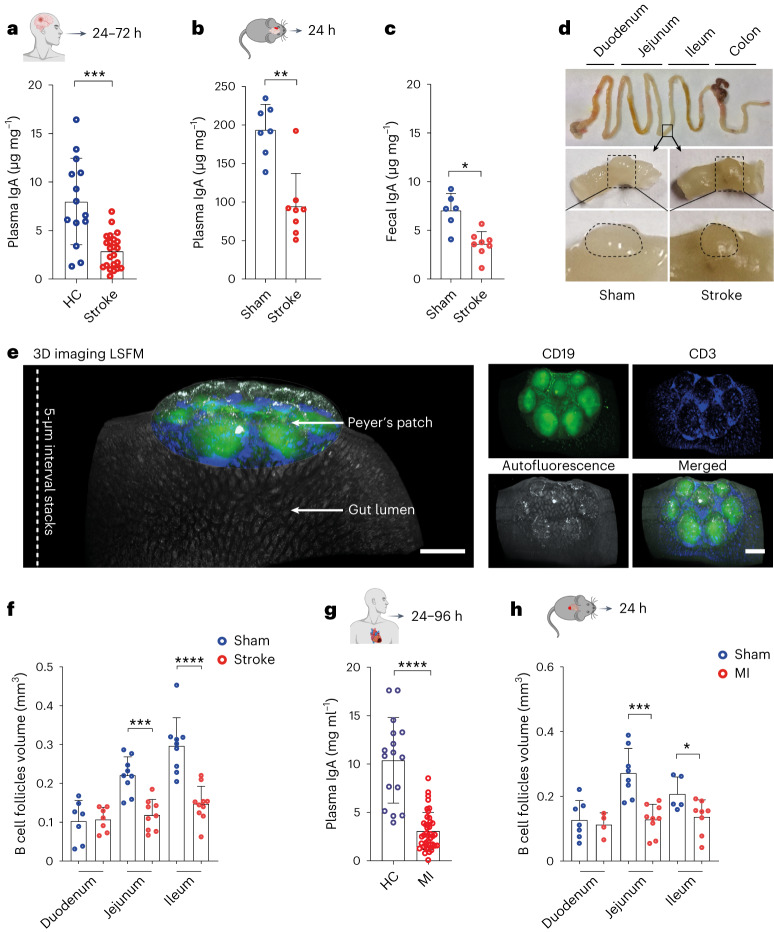
Ischemic stroke and MI reduce plasma IgA levels and B cell follicle volumes in PP. **a**, Concentrations of plasma IgA in patients with stroke and in healthy controls measured by ELISA (*n* = 14−23 per group, *P* = 0.0002). **b**,**c**, Concentrations of plasma IgA and fecal IgA in sham and stroke mice measured by ELISA (*n* = 6−8 per group, **P = 0.037, *P = 0.0053). **d**, Macroscopic overview of the mouse GI tract with the demarcation of PP 1 d after sham surgery or stroke. **e**, Fluorescence images after 3D reconstruction of stained PP showing the position of PP in the small intestine that were whole-mount stained with anti-CD19 (green) and anti-CD3 (blue) fluorescent antibodies before LSFM (left). Fluorescence single−channel images are shown (right); scale bar, 500 μm. **f**, Deep-learning-based automated analysis of B cell follicle volume in PP from duodenum, jejunum and ileum 1 d after stroke or sham (*n* = 7−11 PP per intestinal segment, 4−6 mice per group, sham versus stroke duodenum non-significant *P* > 0.9999, ***P = 0.0003, ****P < 0.0001). **g**, Concentrations of plasma IgA in patients with MI and healthy controls measured by ELISA (*n* = 16−39 per group, ****P < 0.0001). **h**, Deep-learning-based quantification of B cell follicle volume in PP of the duodenum, jejunum and ileum 1 d after sham or myocardial infarction (*n* = 4−9 PP per intestinal segment, 4−5 mice per group, sham versus MI non-significant *P* = 0.7879, ***P = 0.0002, *P = 0.0451). Data are mean ± s.d.; statistical analyses were performed by two-tailed Mann−Whitney *U*-test. All mouse data were combined from at least three independent experiments. HC, healthy control. Source data

**Extended Data Fig. 1:**
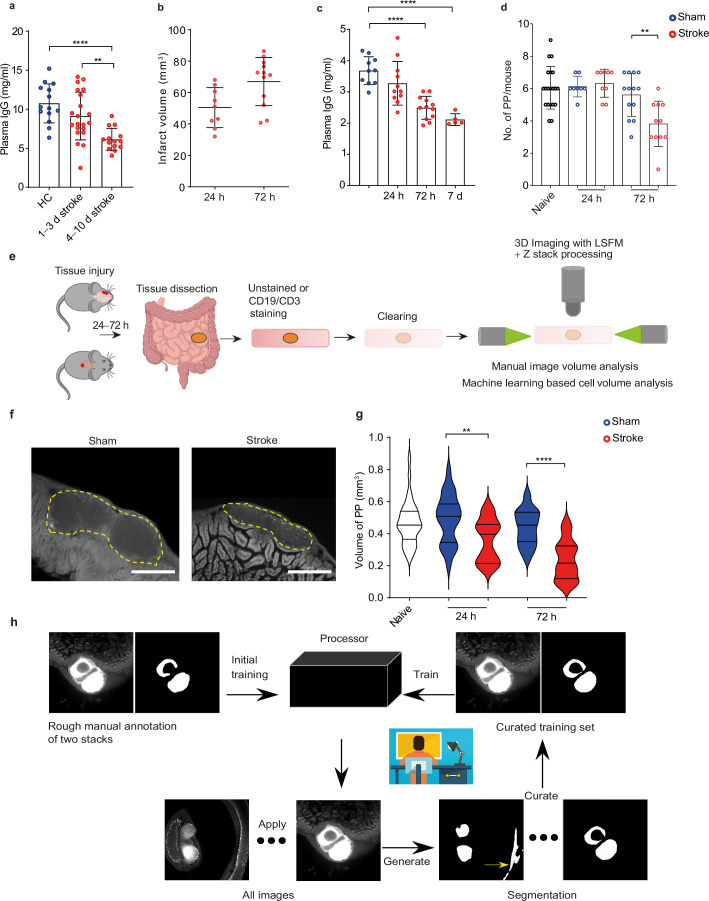
Stroke induces long-term IgG loss in patients and shrinks PP in mice after ischemia-reperfusion injury. **a**, Concentrations of plasma IgG in stroke patients after 1−3 d and 4−10 d of hospital admission and healthy controls measured by ELISA (n = 14−21 per group, ordinary one−way ANOVA, **P = 0.0037, ****P < 0.0001). **b**, Quantification of brain infarct volume after 24 h and 72 h of stroke (n = 9−12 per group). **c**, Quantification of plasma IgG in sham or stroke mice after 24 h, 72 h and 7 d using ELISA (n = 5−12 per group, ordinary one−way ANOVA, ****P < 0.0001). **d**, The total number of intestinal PP per mouse 24 h and 72 h after sham surgery or stroke or unoperated naïve mice (n = 8−13 mice per group, two−tailed Mann−Whitney U test, sham 24 h vs naïve *P* = 0.6883, sham 72 h vs naïve *P* = 0.5810, 24 h sham vs stroke *P* = 0.5597, 72 h sham vs stroke **P = 0.0047). **e**, Illustration of the intestinal tissue preparation for unstained−volume analysis of whole PP or whole−mount staining of B and T cells in PP, followed by tissue clearing and LSFM−based 3D volume analysis. **f**, Representative LSFM images of cleared unstained intestinal PP showing their shrinkage 24 h after sham or stroke, scale bar=500 µm. **g**, Tissue volume analysis of PP after 24 h and 72 h of sham or stroke; (n = 23 PP for sham and n = 16 PP for stroke, n = 3−4 mice for 24 h and n = 34 PP for sham and n = 24 PP for stroke, n = 6 mice for 72 h, two−tailed Mann−Whitney U test, naïve vs sham 24 h *P* = 0.3990, 24 h sham vs stroke **P = 0.0055, 72 h sham vs stroke ****P < 0.0001). **h**, Overview of the human−in−the−loop segmentation workflow for automated analysis of B cell follicles volume and T cell zone volume. Data represented as mean ±s.d., All data are combined from at least three independent experiments. PP=Peyer’s patches.

To determine the amounts of circulating Ig in patients with stroke, we analyzed their plasma samples by immunoassay and revealed a substantial reduction in the circulating amounts of IgA but not IgG within 72 h of hospital admission compared to healthy controls (Fig. 1a and Extended Data Fig. 1a). However, further analysis of a distinct cohort of patients with stroke also showed a delayed (4–10 d after injury) decrease in the amounts of plasma IgG (Extended Data Fig. 1a).

To investigate the mechanisms underlying stroke-induced rapid IgA reduction, we employed a clinically relevant mouse model of ischemic stroke using transient middle cerebral artery occlusion (tMCAO). The induction of brain ischemia resulted in reproducible brain infarcts 1 d and 3 d after injury (Extended Data Fig. 1b). Interestingly, like in patients, we identified a marked reduction in the amounts of plasma and also fecal IgA but not IgG after 24 h in stroke mice compared to sham controls (Fig. 1b,c and Extended Data Fig. 1c). Further longitudinal analyses revealed reduced amounts of plasma IgG at 72 h and 7 d in stroke mice compared to sham controls (Extended Data Fig. 1c).

To find out whether intestinal PPs, which harbor B cell follicles to generate IgA-producing plasma cells, were changed after stroke, we microscopically examined gastrointestinal (GI) tracts of experimental animals. To our surprise, stroke strongly reduced the size of PP compared to sham controls within 24 h of ischemia–reperfusion injury (Fig. 1d). The smaller size of post-stroke PP also led to reduced numbers of harvested PP 72 h after stroke (Extended Data Fig. 1d). To further quantify the extent and cause of post-stroke PP loss, we adapted our three-dimensional (3D) light sheet fluorescence microscopy (LSFM) protocols^12^ and performed a detailed volumetric analysis of PP (Extended Data Fig. 1e). The imaging analysis showed a strong volume reduction (‘melting’) of PP in stroke mice 24 h and 72 h after insult compared to sham controls (Extended Data Fig. 1f,g). However, a retrospective comparison between the volume of PP in sham and naive mice did not show any differences.

**Extended Data Fig. 2:**
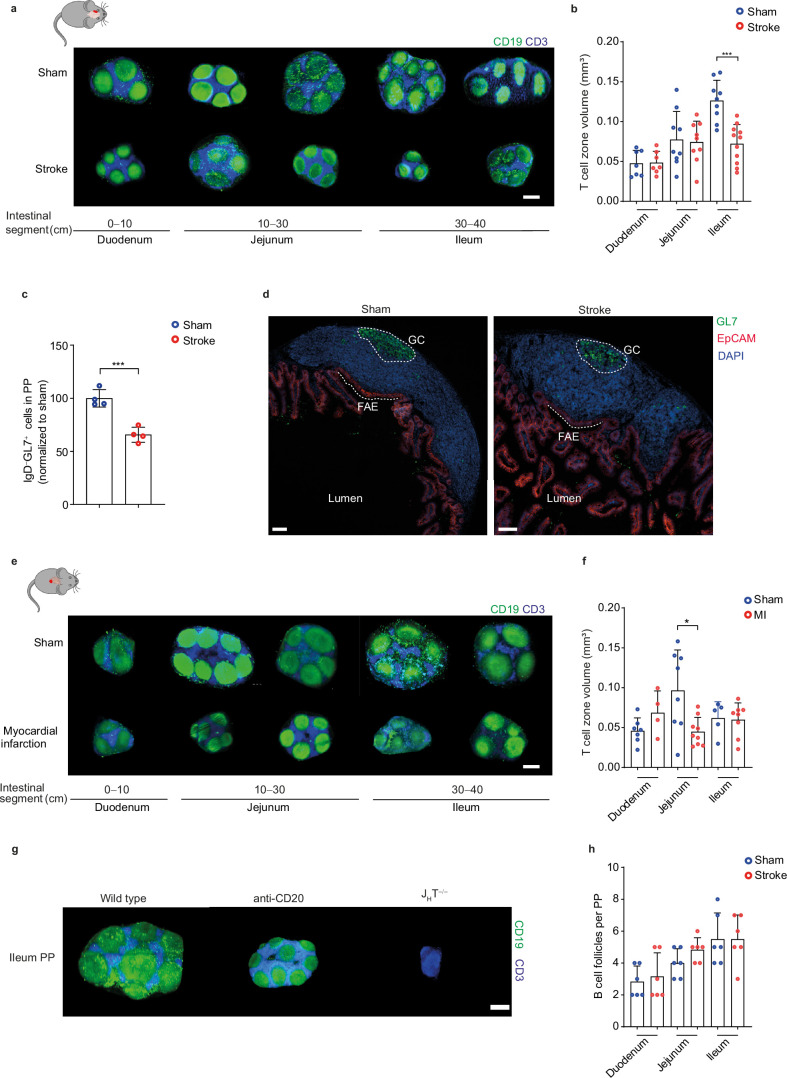
Stroke and myocardial infarction induce shrinkage of B cell follicles and T cell zone volumes in PP. **a**, 3D reconstruction images of CD19^+^ B cells and CD3^+^ T cells in PP isolated from duodenum, jejunum and ileum 24 h after stroke or sham surgery, scale bar=500 µm. **b**, The quantification of T cell zone volume in different intestinal segments after 24 h of stroke or sham controls (n = 8−11 PP per intestinal segment, two−tailed Mann−Whitney U test, duodenum sham vs stroke *P* > 0.9999, jejunum sham vs stroke *P* = 0.8633, leum sham vs stroke ***P = 0.0002). **c**, Numbers of GL7^+^ GC cells in PP after 24 h of sham or stroke operation using flow cytometry (n = 4 mice per group, unpaired t-test ***P = 0.0007). **d**, Representative fluorescence histology images of ileal PP stained with, anti−GL7, anti−EpCAM and DAPI after 24 h of stroke and sham operation, scale bar=200 µm. **e**, 3D reconstruction images of CD19^+^ B cells and CD3^+^ T cells in PP isolated from duodenum, jejunum and ileum 24 h after MI or sham surgery, scale bar=500 µm. **f**, The quantification of T cell zone volume in different intestinal segments after 24 h of myocardial infarction or sham controls (n = 4−6 PP per intestinal segment, two−tailed Mann−Whitney U test, duodenum sham vs MI *P* = 0.1636, jejunum sham vs MI *P = 0.0274, ileum sham vs MI *P* = 0.8329). **g**, 3D reconstruction images of CD19^+^ B cells and CD3^+^ T cells in PP isolated from ileum of naïve wild type, anti-CD20 treated wild type and J_h_T^−/−^ mice, scale bar=500 µm. **h**, Quantification of the number of B cell follicles per PP in different intestinal regions after stroke or sham operation (n = 6 PP per group, two−tailed Mann−Whitney U test, duodenum sham vs stroke *P* = 0.6993, jejunum sham vs stroke *P* = 0.2456, ileum sham vs stroke *P* = 0.4082). Data represented as mean ±s.d., statistical analyses were performed by two−tailed Mann−Whitney U test. All data are combined from at least three independent experiments. PP=Peyer´s patches, MI=myocardial infarction.

To analyze whether PP shrinkage correlates with the loss of B cell follicles, GI tissues were stained with fluorescence-conjugated anti-CD19 and anti-CD3 antibodies and optically cleared before LSFM imaging (Fig. 1e, Extended Data Fig. 1e and Supplementary Video 1). The 3D reconstruction of imaging data showed a strong reduction in the size of CD19^+^ B cell follicles in PP after stroke (Extended Data Fig. 2a). For a thorough and consistent analysis of large numbers of samples, we developed a machine-learning-based 3D volumetric image analysis (Extended Data Fig. 1h). We found that PP of stroke mice exhibited smaller volumes of CD19^+^ B cell follicles in the jejunum and ileum compared to similar regions in sham controls (Fig. 1f and Supplementary Video 2). In addition, stroke caused a considerable reduction of CD3^+^ T cell zone volumes in PP isolated from the ileum (Extended Data Fig. 2b). Further microscopy and flow cytometry data confirmed that, within 24 h, stroke instigated a reduction in GL7^+^ GC cells (Extended Data Fig. 2c) yet with no structural disruption of GC or the follicular-associated epithelium (FAE) (Extended Data Fig. 2d).

To identify whether these findings were specific to stroke or, rather, a global response to large-scale tissue injury, we studied circulating levels of IgA in patients with MI. Interestingly, in addition, patients with MI presented reduced amounts of plasma IgA (Fig. 1g). To clarify whether the reduced plasma IgA amounts in patients with MI were also related to the shrinkage of PP, we used a murine model of myocardial ischemia–reperfusion injury^13^. MI caused the shrinkage of PP B cell follicles in the intestinal jejunum and ileum and T cell zone volumes in the jejunum as compared to sham controls (Fig. 1h and Extended Data Fig. 2e,f).

Of note, a specific absence of B cells in J_H_T^−/−^ mice^14^ or their depletion using anti-CD20 antibodies reduced PP size, thus highlighting a B cell role in maintaining PP structure (Extended Data Fig. 2g). Collectively, these results showed a strong effect of sterile tissue injury on the rapid and massive loss of PP-resident B and T cells without affecting the average numbers of detectable individual B cell follicles in the affected PP (Extended Data Fig. 2h).

## Stroke shortens the survival of intestinal immature and mature plasma cells

**Fig. 2:**
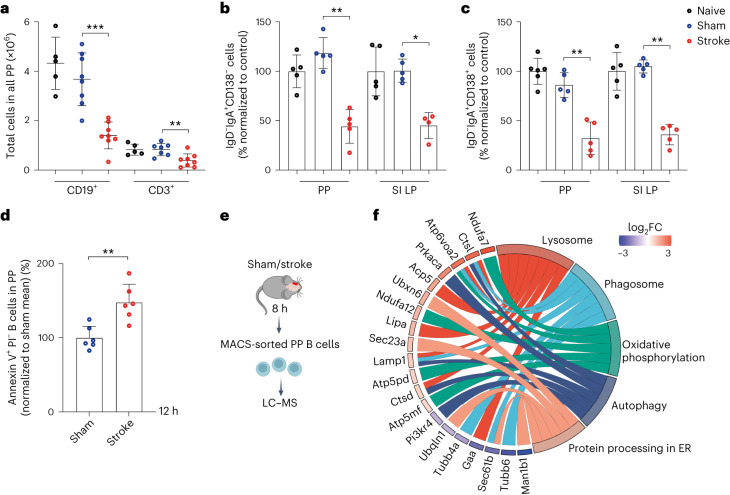
Stroke induces B cell loss in PP via activation of cell death pathways. **a**, Flow-cytometry-based quantification of the number of CD19^+^ B cells and CD3^+^ T cells in all intestinal PP 24 h after sham surgery or stroke and unoperated naive mice (*n* = 5−8 per group, sham versus naive non-significant *P* = 0.4351, ***P = 0.0003, **P = 0.0037)**. b**, Quantification of IgD^−^IgA^+^CD138^−^ IgA-switched B cells in all PP and SI LP after 24 h of sham surgery or stroke and naive mice (*n* = 5 per group, naive versus sham PP non-significant *P* = 0.2222, sham versus stroke PP **P = 0.0079, naive versus sham SI LP non-significant *P* = 0.8413, sham versus stroke SI LP *P = 0.0159). Data are presented as a percentage change and normalized to the mean of naive controls **c**, Quantification of IgD^−^IgA^+^CD138^+^ plasma cells in all PP and SI LP (*n* = 5 per group, naive versus sham PP non-significant *P* = 0.1775, sham versus stroke PP **P = 0.0079, naive versus sham SI LP non-significant *P* = 0.5476, sham versus stroke SI LP **P = 0.0079). **d**, Single-cell suspensions from PP of sham and stroke mice were prepared 12 h after the operation, and apoptotic cells were quantified by staining with Annexin V and PI followed by flow cytometry analysis (*n* = 6 per group, **P = 0.0043). **e**, After sham operation or stroke, B cells from PP were enriched using MACS and analyzed by mass spectrometry (*n* = 3 per group). **f**, Gene Ontology chord diagram of the functional enrichment analysis. Genes are ranked based on log_2_FC value (high to low). Each chord connects the gene with its associated pathway. Data are mean ± s.d.; statistical analyses were performed by two-tailed Mann−Whitney *U*-test. All data were combined from at least three independent experiments. ER, endoplasmic reticulum.

**Extended Data Fig. 3:**
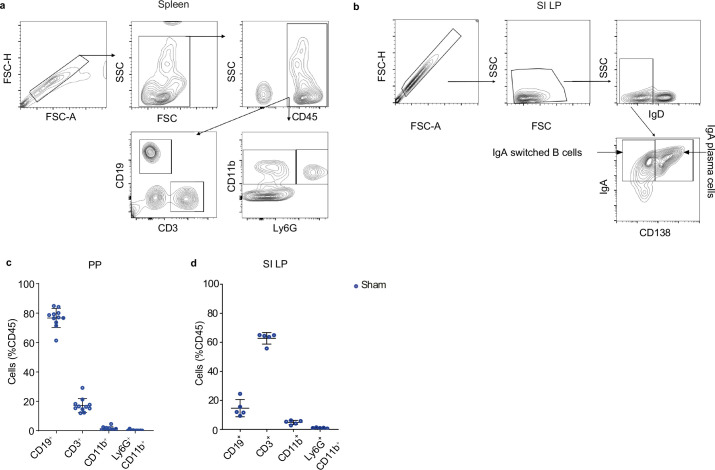
Differential frequencies of B and T cells in PP and SI LP. **a**, Representative gating strategy for the analysis of lymphocytes and granulocytes in spleen using multi−color flow cytometry. **b**, Representative gating strategy for the analysis of IgA-switched B cells and IgA^+^ mature plasma cell subsets in SI LP using multi-color flow cytometry. **c**, The frequency of CD19^+^, CD3^+^, CD11b^+^ and Ly6G^+^ CD11b^+^ cells in PP (n = 11 mice per group) and **d**, SI LP (n = 5 mice per group) of sham mice. Data represented as mean ±s.d. All data are combined from at least three independent experiments. PP=Peyer´s patches, SI LP=small intestine lamina propria.

**Extended Data Fig. 4:**
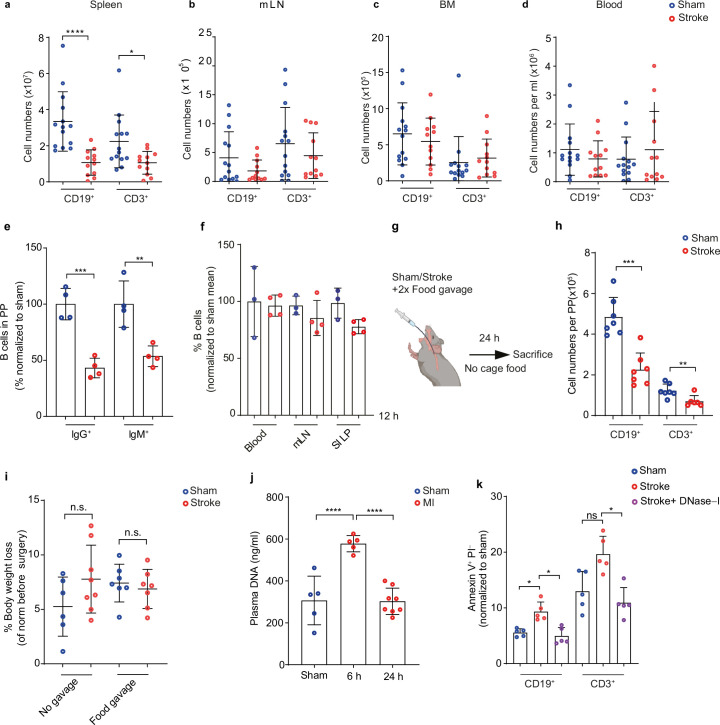
Stroke differentially impacts lymphocyte numbers in lymphoid tissues and is independent of reduced food consumption. **a**, Total numbers of CD19^+^ B cells and CD3^+^ T cells in spleens of stroke or sham−operated mice (n = 12−14 per group, two−tailed Mann−Whitney U test, ****P < 0.0001, *P = 0.0202). **b-d**, Total numbers of CD19^+^ B cells and CD3^+^ T cells in mLN (n = 12−14 per group, two−tailed Mann−Whitney U test, CD19^+^ cells *P* = 0.3744, CD3+ cells *P* = 0.5952), BM (n = 12−14 per group, two−tailed Mann−Whitney U test, CD19^+^ *P* = 0.6308, CD3^+^ *P* = 0.3217) and blood (n = 12−14 per group, two−tailed Mann−Whitney U test, CD19^+^ *P* = 0.5267, CD3^+^ *P* = 0.7045) of stroke or sham−operated mice. **e**, The percentage change in IgG^+^ and IgM^+^ B cells in PP after 24 h of sham or stroke operation (n = 4 mice per group, unpaired t-test, ***P = 0.0005, **P = 0.0064). Data is presented as normalized to the mean of sham. **f**, Relative frequencies of B cells in blood, mLN and SI LP after 12 h of stroke or sham−operation (n = 3−4 per group) normalized to mean of sham. **g**, Mice underwent stroke or sham surgery and received two times food gavage and were sacrificed 24 h after the operation to analyze the number of B and T cells in PP. **h**, Total numbers of CD19^+^ B cells and CD3^+^ T cells in PP of stroke or sham−operated mice that received food gavage (n = 7 per group, two−tailed Mann−Whitney U test, ***P = 0.0006, **P = 0.0023). **i**, Percentage of body-weight loss in sham and stroke mice with and without food gavage (n = 6−8 per group, two−tailed Mann−Whitney U test, no gavage sham vs stroke *P* = 0.2284, food gavage sham vs stroke *P* = 0.5589). **j**, Quantification of plasma DNA content in sham−operated or MI mice (n = 5−8 per group, ordinary one−way ANOVA, ****P < 0.0001). **k**, Cell cultures from PP were prepared and treated with the plasma of sham or stroke mice for 12 h. In the third condition, plasma of stroke mice was treated with DNase−I before addition to the cell cultures and quantification of Annexin V^+^ PI^−^ cells was performed by flow cytometry (n = 5 per group). Data represented as mean ±s.d., Shapiro-Wilk normality test. PP=Peyer’s patches, SI LP=small intestine lamina propria, mLN=mesenteric lymph nodes, MI=myocardial infarction.

We further investigated different leukocyte populations in PP, small intestine lamina propria (SI LP), mesenteric lymph nodes (mLNs), bone marrow (BM) and blood using flow cytometry (Extended Data Fig. 3a,b). Most immune cells in PP were CD19^+^ B (78 ± 10%) and CD3^+^ T (18 ± 5%) cells, with a small fraction of CD11b^+^Ly6G^−^ monocytes (<2%) and Ly6G^+^CD11b^+^ neutrophils (<1%) (Extended Data Fig. 3c). However, SI LP showed reverse frequencies of CD19^+^ B (18 ± 4%) and CD3^+^ T (61 ± 4%) cells (Extended Data Fig. 3d). As revealed in our ultramicroscopic analysis, stroke strongly reduced the total numbers of CD19^+^ B cells and CD3^+^ T cells in PP after 24 h (Fig. 2a). The absence of lymphocyte loss in sham mice compared to naive controls validated the contribution of stroke on the massive loss of B and T cells in PP. In keeping with our previous data^15,16^, stroke also strongly decreased the numbers of B and T cells in spleen compared to controls (Extended Data Fig. 4a). However, B and T cells in mLNs, BM and blood were not significantly reduced (Extended Data Fig. 4b–d).

Further analyses found a reduction in IgA-switched B cells (IgD^−^IgA^+^CD138^−^) in total PP and SI LP in stroke mice compared to sham controls (Fig. 2b). In PP, B cells are primed by signals from the intestinal microflora and other immune cells to produce IgA^+^-secreting plasma cells for their final homing to LP^17^. Our data showed that stroke substantially decreased IgD^−^IgA^+^CD138^+^ plasma cells in PP and SI LP (Fig. 2c). The finding that stroke also reduced IgG^+^ and IgM^+^ B cells in PP suggested generalized survival defects in Ig-secreting cells (Extended Data Fig. 4e). Next, we performed different in vivo experiments to delineate the mechanisms of B cell loss in PP after stroke. Interestingly, the induction of stroke increased the percentage of Annexin V^+^ PI^−^ apoptotic B cells in PP within 12 h compared to sham controls (Fig. 2d). To elucidate a possible active egress of B cells from PP after stroke, we consistently analyzed other lymphoid tissues 12 h after surgery. However, our data did not show any increase in the percentages of B cells in blood, mLN and SI LP compared to sham controls (Extended Data Fig. 4f). Hence, enhanced B cell egress is unlikely to explain the massive PP shrinkage after stroke.

To study the impact of stroke on the molecular makeup of PP B cells, we performed mass-spectrometry-based proteomics analyses after 8 h (Fig. 2e). Out of 3,997 identified total proteins in B cells, 99 proteins were upregulated and 86 were downregulated in B cells isolated from stroke compared to sham-treated animals. Interestingly, functional enrichment analyses of differentially expressed proteins showed dysregulation of pathways associated with lysosome and phagosome function, autophagy, oxidative phosphorylation and the endoplasmic reticulum (Fig. 2f). Autophagy-associated proteins, such as Catsl and Lamp1, were upregulated in B cells from stroke mice. Moreover, the upregulated proteins Ndufa7 and Atp6voa2 are involved in oxidative phosphorylation. This can be a response to DNA damage and cell stress because Atp6voa2 is specifically known to be an essential factor for autophagy, and Ndufa7 is associated with reactive oxygen species (ROS) detoxification. Overall, these data indicate that stroke may trigger specific B cell stress pathways and possibly excessive autophagy-mediated cell death^18^.

## Long-term metabolic dysfunction in B cells after stroke

**Fig. 3:**
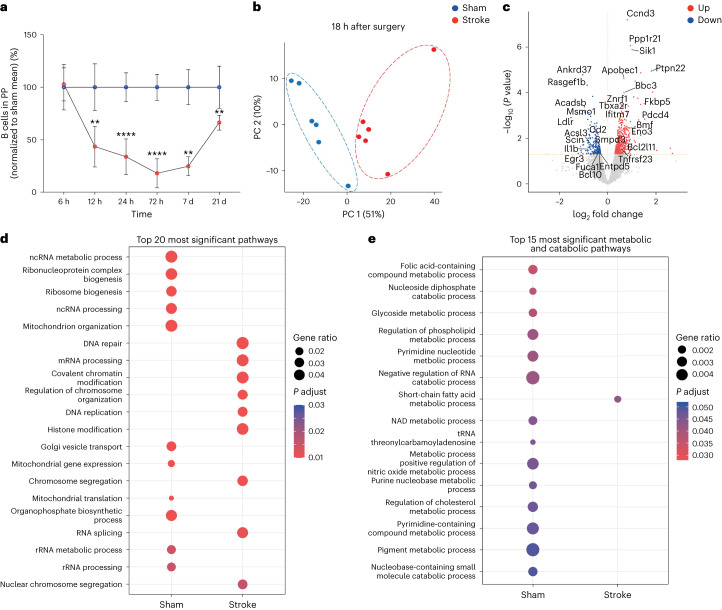
Stroke downregulates cellular and metabolic functional gene pathways of B cells in PP. **a**, Kinetics of B cell loss in PP of stroke mice compared to sham controls. Data are presented as a percentage decrease in B cell numbers in stroke mice normalized to mean of sham controls for each timepoint (*n* = 6−9 per group and timepoint, sham versus stroke 6 h *P* = 0.9372, 12 h **P = 0.079, 24 h ****P < 0.0001, 72 h ****P < 0.0001, 7 d **P = 0.0095, 21 d **P = 0.0087). **b**, PCA of RNA-seq data of CD19^+^ B cells in PP from mice exposed to sham surgery or stroke (*n* = 6 per group). **c**, Volcano plot showing statistically significant differentially expressed genes in PP B cells of stroke mice compared to sham, as determined by non-multiple-testing-adjusted *P* values from two-sided Wald test. Red dots indicate upregulated genes, and blue dots indicate downregulated genes. **d**, GSEA of RNA-seq showing enriched cell function pathways in sham-operated and stroke mice as determined by one-sided Fisher’s exact test *P* values, adjusted for multiple testing with FDR and significance set at *P* < 0.05. **e**, GSEA showing enriched metabolic**/**catabolic pathways in PP B cells of stroke and sham-operated mice. The dot size indicates the calculated gene ratio, and the dot color indicates the adjusted *P* value representing the enrichment score as described in the Methods. Note that B cells from stroke mice appear metabolically inactive. Data represent mean ± s.d., two-tailed Mann−Whitney *U*-test. All data were combined from at least three independent experiments, *n* = 6 mice per group. Down, downregulated genes; Up, upregulated genes.

We next aimed to investigate the duration of B cell loss from PP after stroke, which is therapeutically relevant to identify a potential need to treat prolonged intestinal immune imbalance. Our data showed a decrease in B cell numbers after 12 h, which persisted at least until 7 d in stroke mice compared to sham controls (Fig. 3a). As late as 21 d after stroke, B cell numbers in PP showed partial recovery but were still significantly lower than sham controls. To reveal the underlying mechanisms of this long-term loss of cells, we purified residual PP B cells from mice 18 h after sham operation or stroke and analyzed transcriptomic changes by bulk RNA sequencing (RNA-seq) and bioinformatic analyses. Interestingly, a principal component analysis (PCA) showed that the transcriptomes of B cells clustered separately in stroke mice and sham controls (PC1, at 51%) (Fig. 3b). Moreover, 447 genes were upregulated and 198 genes were downregulated (absolute fold change (FC) ≥ 2 and adjusted *P* < 0.05) in B cells after stroke compared to sham controls (Fig. 3c). Further supporting the proteomics data, cell death genes (*Bmf*, *Pdcd4*, *Bbc3* and *Znrf1*) were enriched while cell function genes (*Egr3*, *Ankrd37* and *Rasgef1b*) and cell metabolic genes (*Fuca-1*, *Smpd3*, *Acsl3*, *Ldlr* and *Entpd5*) were reduced in B cells of stroke mice compared to sham controls (Fig. 3c).

B cells from stroke mice expressed higher levels of the Myc target gene cyclin *Ccnd3* and B cell lymphoma *Bcl2*, a key regulator of clonal expansion and survival, respectively. In addition, stroke enriched the expression of *Sik1* and *Ptpn22* in B cells, which are involved in cell differentiation and B cell receptor signaling. Furthermore, we performed gene set enrichment analyses (GSEAs) to identify cellular processes that were affected in B cells after stroke. The differentially expressed genes enriched in B cells of stroke mice were related to chromatin and histone remodeling, DNA replication and repair (Fig. 3d). Notably, the pathways responsible for mitochondrial organization and function, ribosome biogenesis and Golgi vesicle transport were decreased in B cells of stroke mice, suggesting severe metabolic disturbances in these cells. Further analyses showed a complete downregulation of pathways related to several metabolic and catabolic processes in B cells after stroke compared to sham controls (Fig. 3e).

Next, to exclude that suboptimal food supply after stroke compared to sham was responsible for B cell death and PP shrinkage, stroke and sham mice were gavaged with liquid food and euthanized 24 h after ischemia–reperfusion injury (Extended Data Fig. 4g). The analysis showed that stroke still reduced lymphocyte numbers in PP compared to sham controls with similar body weights, thus excluding a potential impact of reduced food intake after surgery on PP melting (Extended Data Fig. 4h,i).

## Circulating DNA triggers lymphocyte reduction in intestinal tissues

**Fig. 4:**
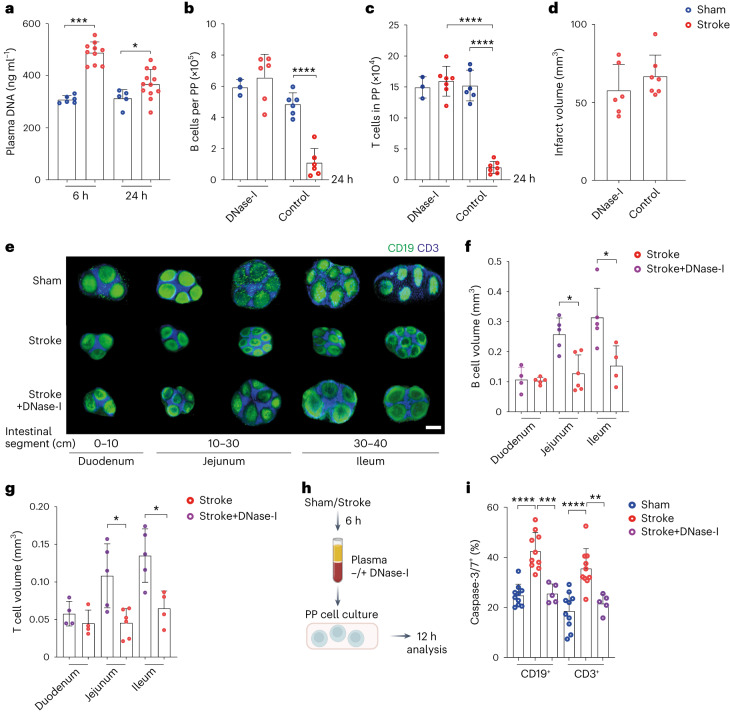
Stroke increases ciDNA and promotes lymphocyte loss in PP. **a**, Quantification of plasma DNA 6 h and 24 h after sham surgery or stroke using Qubit assays (*n* = 5−12 per group, two−tailed Mann−Whitney *U*-test, ***P = 0.0002, *P = 0.0459). **b**, Numbers of B cells in PP 24 h after sham surgery or stroke in DNase-I-treated and vehicle-treated mice analyzed by flow cytometry (*n* = 3−6 per group, ordinary one-way ANOVA with Bonferroni’s multiple comparisons tests, sham+DNase-I versus stroke+DNase-I non-significant *P* > 0.9999, sham control versus sham+DNase-I non-significant *P* > 0.9999, ****P < 0.0001). **c**, Numbers of T cells in PP 24 h after sham operation or stroke in DNase-I-treated and vehicle-treated mice (*n* = 3−7 per group, ordinary one-way ANOVA with Bonferroni’s multiple comparisons tests, sham+DNase-I versus stroke+DNase-I non-significant *P* > 0.9999, sham control versus stroke control ****P < 0.0001, stroke control versus stroke+DNase-I ****P < 0.0001). **d**, Brain infarct volumes in DNase-I-treated and untreated mice at 24 h (*n* = 6−7 per group, two−tailed Mann−Whitney *U*-test, non-significant *P* = 0.2343). **e**, 3D reconstruction LSFM images of CD19^+^ B cells and CD3^+^ T cells in PP after sham, stroke and stroke+DNase-I-treated mice; scale bar, 500 µm. **f**, Quantification of CD19^+^ B cell follicle volume (*n* = 4−6 PP per intestinal segment, two-tailed Mann−Whitney *U*-test, stroke versus stroke+DNase-I duodenum non-significant *P* = 0.9048, stroke versus stroke+DNase-I jejunum *P = 0.0173, stroke versus stroke+DNase-I ileum *P = 0.0317). **g**, T cell zone volume in duodenum, jejunum and ileum 24 h after stroke or stroke+DNase-I treatment (*n* = 4−6 PP per intestinal segment, two-tailed Mann−Whitney *U*-test, stroke versus stroke+DNase-I duodenum non-significant, *P* = 0.2, stroke versus stroke+DNase-I jejunum *P = 0.0173, stroke versus stroke+DNase-I ileum *P = 0.0317). **h**, Schematic of the experimental paradigm. Mice underwent stroke or sham operation and were euthanized after 6 h to collect blood plasma. Cell cultures from PP were prepared and treated with the plasma of sham or stroke mice for 12 h. In the third condition, plasma of stroke mice was treated with DNase-I before addition to the cell cultures, and quantification of caspase-3/7-expressing cells was performed by flow cytometry. **i**, The percentages of caspase-3/7^+^ B and T cells in PP cell cultures incubated with the plasma of sham or stroke mice or DNase-I-treated stroke plasma (*n* = 5−10 mice per group, ordinary one-way ANOVA, CD19^+^ sham versus stroke ****P < 0.0001, stroke versus stroke+DNase-I ***P = 0.0002, CD3^+^ sham versus stroke ****P < 0.0001, stroke versus stroke+DNase-I **P = 0.0037). Data are mean ± s.d., Shapiro–Wilk normality tests.

To elucidate the mechanisms of B cell loss in PP, we focused on our earlier concept of soluble DNA as a death mediator released after tissue injury^15^. We found an early (6 h) increase in circulating DNA (ciDNA) in stroke mice compared to controls (Fig. 4a). Similarly, MI increased the levels of ciDNA at 6 h, which, however, returned to sham levels at 24 h (Extended Data Fig. 4j). To test a potential causative role of ciDNA for B cell loss in PP, we treated the mice with recombinant DNase-I immediately after stroke. Interestingly, the degradation of ciDNA substantially inhibited the loss of PP B cells (Fig. 4b) and T cells (Fig. 4c). Moreover, DNase-I treatment did not affect brain infarct volumes (Fig. 4d) at this early timepoint of 24 h after injury, thus indicating a direct blockage of B cell loss with DNase-I and ciDNA acting as a soluble mediator released after stroke.

Further LSFM analysis indeed confirmed that DNase-I treatment after stroke completely preserved PP structural integrity (Fig. 4e) and the volume of B and T cell compartments in the affected intestinal segments (Fig. 4f,g). To prove the contribution of plasma components as a cause of lymphocyte death, we used plasma of sham or stroke mice (6 h after injury) for further ex vivo analyses and incubated it with PP cell cultures (Fig. 4h). The treatment of PP cells with the plasma of stroke mice increased the percentage of caspase-3/7^+^ and Annexin V^+^ PI^−^ B and T cells, which was abrogated after the incubation of stroke plasma with DNase-I, supporting the principal ability of circulating mediators to directly induce cell death (Fig. 4i and Extended Data Fig. 4k).

## Neutrophils are the major source of ciDNA after stroke

Next, we explored the potential sources of the additional amounts of ciDNA after stroke. Activated neutrophils are the first intruders to injured inflammatory tissues and respond via the release of NETs^19^.

**Fig. 5:**
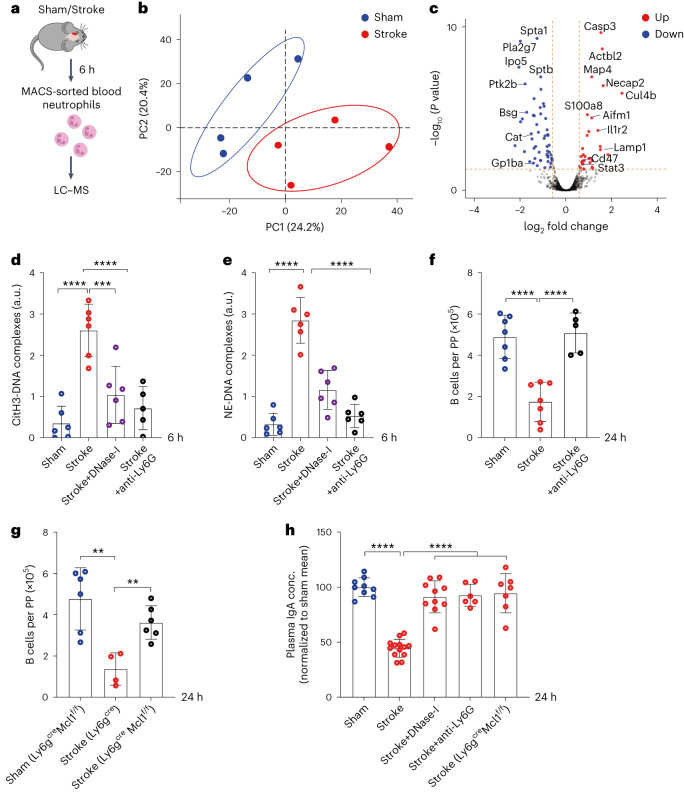
Stroke activates circulating neutrophils to release cytotoxic NETs, which induce B cell loss. **a**, Schematic of the experimental paradigm for neutrophil mass spectrometry and proteomics analysis. Blood neutrophils were isolated 6 h after sham surgery or stroke to perform liquid chromatography–mass spectrometry analysis. **b**, PCA of neutrophil proteomics after sham or stroke (*n* = 4 mice per group). **c**, Volcano plot comparing the normalized protein abundance in blood neutrophils of stroke mice versus sham-operated mice. Red dots indicate significantly upregulated proteins, and blue dots indicate significantly downregulated proteins. Two-tailed *t*-test, *P* < 0.05 adjusted with the Benjamini–Hochberg method, FC > 1.5 or FC < 0.5. **d**, Relative plasma levels of citH3-DNA (*n* = 5−6 per group, sham versus stroke ****P < 0.0001, stroke versus stroke+DNase-I ***P = 0.0004, stroke versus stroke+anti-Ly6G ****P < 0.0001). **e**, NE-DNA complexes after sham+isotype antibody, stroke+isotype antibody, stroke+DNase-I treatment or stroke+anti-Ly6G antibody treatment (*n* = 5−6 per group, sham versus stroke, stroke versus stroke+DNase-I, stroke versus stroke+anti-Ly6G treatment ****P < 0.0001). **f**, Numbers of CD19^+^ B cells in intestinal PP in sham-operated+isotype antibody, stroke+isotype antibody and stroke+anti-Ly6G antibody-treated mice (*n* = 5−7 per group, sham versus stroke, stroke versus stroke+anti-Ly6G treatment ****P < 0.0001). **g**, Numbers of CD19^+^ B cells in PP in sham-operated Ly6g^cre^Mcl1^f/f^ mice, stroke Ly6g^cre^ and Ly6g^cre^Mcl1^f/f^ mice (*n* = 4−6 per group, sham versus Ly6g^cre^ **P = 0.0013, stroke Ly6g^cre^ versus stroke Ly6g^cre^Mcl1^f/f^ **P = 0.0095). **h**, Quantification of plasma IgA amounts sham, stroke, stroke+DNase-I treatment, stroke+anti-Ly6G antibody treatment or stroke Ly6g^cre^Mcl1^f/f^ mice (*n* = 6−13 per group, stroke versus sham, stroke versus stroke+DNase-I, stroke versus stroke+anti-Ly6G antibody, stroke versus stroke Ly6g^cre^Mcl1^f/f^ ****P < 0.0001). Data represent mean ± s.d., Shapiro−Wilk normality and ordinary one-way ANOVA with Bonferroni’s multiple comparisons tests.

**Extended Data Fig. 5:**
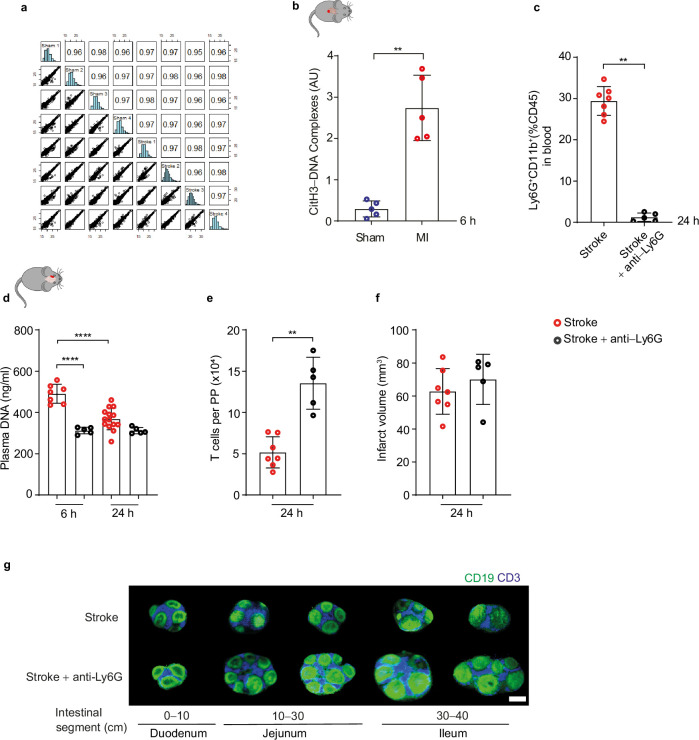
Neutrophil-released NETs after tissue injury contribute to reduced B and T cell numbers in PP. **a**, A sample-wise comparison showed a Pearson correlation coefficient for all analyzed samples for mass spectrometry analysis. **b**, Relative plasma levels of citH3−DNA complexes after 6 h of myocardial infarction or sham operation (n = 5 per group, two−tailed Mann−Whitney U test, **P = 0.0079). **c**, Relative frequencies of blood Ly6G^+^CD11b^+^ neutrophils in stroke mice treated with anti−Ly6G antibodies or vehicle (n = 5−7 per group, two−tailed Mann−Whitney U test, **P = 0.0025). **d**, Quantification of plasma DNA at 6 h and 24 h after stroke + isotype antibody or stroke + anti−Ly6G antibody injected mice (n = 5−14 per group, ordinary one−way ANOVA, ****P < 0.0001, Ly6G antibody 6 h vs 24 h *P* > 0.9999). **e**, The total numbers of CD3^+^ T cells in PP of stroke + isotype antibody or stroke + anti−Ly6G antibody−injected mice after 24 h (n = 5−7 per group, two−tailed Mann−Whitney U test, **P = 0.0025). **f**, Brain infarct volumes after 24 h of stroke + isotype antibody or stroke + anti−Ly6G antibody−injected mice (n = 5−7 per group, two−tailed Mann−Whitney U test, *P* = 0.3434)**. g**, 3D reconstruction images of CD19^+^ B cells and CD3^+^ T cells in PP isolated from duodenum, jejunum and ileum 24 h of stroke + isotype antibody or stroke + anti−Ly6G treated mice, scale bar=500 μm. Data represented as mean ±s.d., Shapiro-Wilk normality. PP=Peyer’s patch, CitH3=citrullinated Histone-3.

To find out whether circulating neutrophils in stroke mice were activated, we characterized their molecular makeup 6 h after sham surgery or stroke using mass spectrometry (Fig. 5a). The comparative and label-free quantitative proteomic analysis yielded 1,757 proteins with two or more unique peptides and 1% false discovery rate (FDR). A sample-wise comparison showed a Pearson correlation coefficient of ≥0.93, indicating high technical reproducibility (Extended Data Fig. 5a). Interestingly, the PCA showed a clear separation between circulating neutrophils of sham and stroke mice (Fig. 5b) based on their proteome. Of the 89 significantly altered proteins (adjusted *P* < 0.05 and FC ≥ 1.5), 32 were upregulated and 57 were downregulated in neutrophils of stroke mice compared to sham controls (Fig. 5c). Remarkably, proteins associated with neutrophil degranulation (Lamp1, S100a8 and CD47) and neutrophil activation (Il1r2 and Stat3) were upregulated in stroke mice. In addition, the increased abundance of apoptotic proteins (Aifm1 and Casp3) and the deficiency of cell function proteins (Pla2g7, Spta1, Sptb, Ipo5, Bsg, Gp1ba and Ptk2b) in neutrophils from stroke mice indicated the activation of cell death pathways that have been observed in association with chromatinolysis^20^ and NETosis^21^.

**Extended Data Fig. 6:**
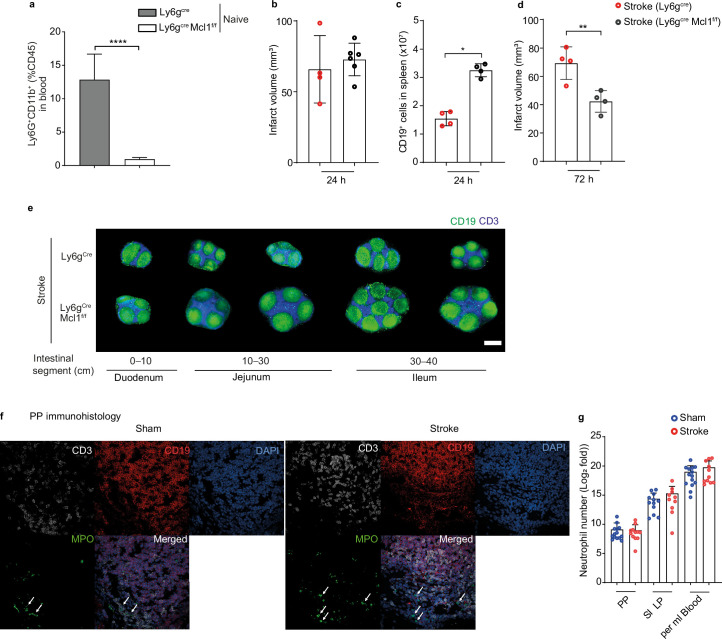
Neutrophil−deficiency blocked stroke triggered lymphocyte loss and shrinkage of B cell follicles in PP. **a**, Percentages of blood Ly6G^+^ CD11b^+^ neutrophils in naïve Ly6g^cre^ and Ly6g^cre^Mcl1^f/f^ mice (n = 11−15 per group, ****P < 0.0001). **b**, Brain infarct volumes after 24 h of stroke in Ly6g^cre^ and Ly6g^cre^Mcl1^f/f^ mice (n = 4−6 per group, *P* = 0.4762). **c**, Total numbers of CD19^+^ T cells after 24 h of stroke in spleens of Ly6g^cre^ and Ly6g^cre^Mcl1^f/f^ mice (n = 4 per group, *P = 0.0286)**. d**, Brain infarct volumes after 72 h of stroke in Ly6g^cre^ and Ly6g^cre^Mcl1^f/f^ mice (n = 4 per group, **P = 0.008). **e**, 3D reconstruction images of CD19^+^ B cells and CD3^+^ T cells in PP isolated from duodenum, jejunum and ileum 24 h after stroke in Ly6g^cre^ and Ly6g^cre^Mcl1^f/f^ mice, scale bar=500 μm. **f**, Representative fluorescence histology images of PP stained with, anti−CD3, anti−CD19, anti−MPO and DAPI after 12 h of stroke and sham operation, scale bar=20 µm. **g**, Log^2^ fold numbers of Ly6G^+^ CD11b^+^ neutrophils in PP, SI LP and blood in sham or stroke mice (n = 12−14 mice per group, PP comparison *P* = 0.8201, SI LP comparison *P* = 0.4562, per ml Blood comparison *P* = 0.4668). Data represented as mean ±s.d., statistical analyses were performed by two−tailed Mann−Whitney U test. PP=Peyer’s patches, SI LP=small intestine lamina propria.

To determine whether NETs were indeed released into the circulation after stroke, we measured the plasma content of citrullinated histone H3 (citH3)-DNA and neutrophil elastase (NE)-DNA complexes, both hallmarks of NETosis^22^. Indeed, 6 h after stroke or MI, we observed increased levels of citH3-DNA and NE-DNA complexes in the blood (Fig. 5d,e and Extended Data Fig. 5b). Interestingly, treatment of mice with DNase-I immediately after stroke strongly reduced the levels of citH3-DNA and NE-DNA complexes (Fig. 5d,e). To further validate the contribution of neutrophils to NET release and B cell loss in PP after stroke, we applied our established dual antibody-mediated depletion of circulating neutrophils (Extended Data Fig. 5c)^23^. Indeed, neutrophil removal before the induction of stroke substantially reduced citH3-DNA and NE-DNA complexes (Fig. 5d,e) and total ciDNA (Extended Data Fig. 5d) and also completely inhibited the loss of B and T cells in PP (Fig. 5f and Extended Data Fig. 5e), without affecting the brain infarct volumes (Extended Data Fig. 5f). In addition, neutrophil depletion inhibited the stroke-induced shrinkage of B cell follicles in PP (Extended Data Fig. 5g). Acutely depleting neutrophils by antibody injection might have uncontrolled secondary effects on immune homeostasis. Hence, we used neutropenic Ly6g^cre^Mcl^f/f^ mice that genetically lack the critical survival factor Mcl specifically in neutrophils^24^. Confirming our antibody-depletion experiments, mice with genetically induced neutropenia (Extended Data Fig. 6a) showed higher numbers of B cells in PP compared to neutrophil-sufficient Ly6g^cre^ mice 24 h after stroke (Fig. 5g) without experiencing different infarct volumes (Extended Data Fig. 6b). Moreover, neutrophil-deficient Ly6g^cre^Mcl^f/f^ mice also showed increased numbers of splenic B cells compared to Ly6g^cre^ controls after stroke (Extended Data Fig. 6c), thus suggesting a main role of neutrophils for augmented ciDNA generation and B cell apoptosis induction. Because activated neutrophils exhibit neurotoxic functions^25,26^, their deficiency in Ly6g^cre^Mcl^f/f^ mice also showed reduced brain infarcts after 3 d of stroke (Extended Data Fig. 6d). In addition, when stroke mice were depleted of neutrophils, we found not only normalized levels of plasma IgA (Fig. 5h) but also a substantial inhibition of PP shrinkage in all studied intestinal segments (Extended Data Fig. 6e). Because we found only small numbers of MPO^+^ or Ly6G^+^ neutrophils directly within the PP of stroke mice (Extended Data Fig. 6f,g), we assume that acute NET production by circulating neutrophils in response to sterile injury is the major trigger that leads to PP lymphocyte cell death.

**Fig. 6:**
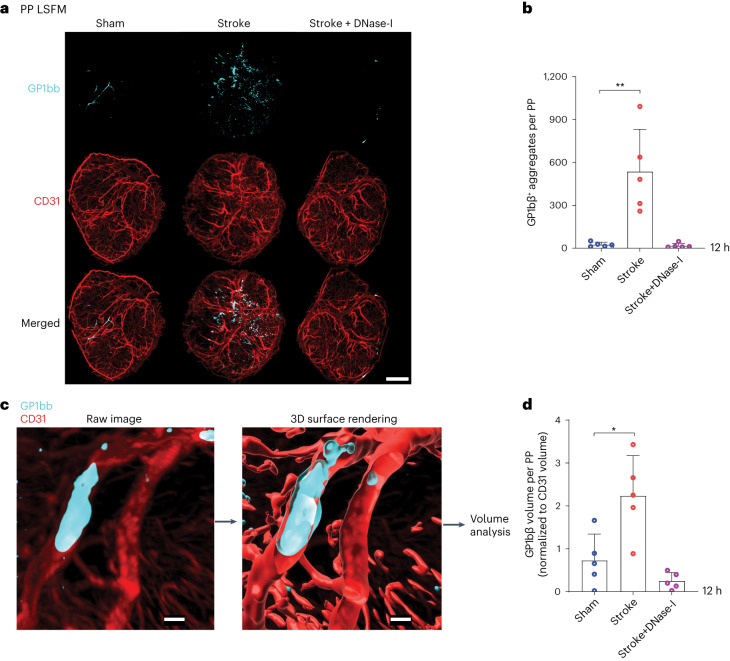
Stroke triggers platelet aggregation and vascular thrombus formation in PP. **a**, 3D reconstruction images of GP1bβ^+^ platelet aggregates in CD31^+^ vasculature in PP isolated from sham, stroke+vehicle and stroke+DNase-I-treated mice. Scale bar, 300 µm. **b**, Quantification of GP1bβ^+^ platelet aggregates in PP isolated from sham, stroke+vehicle and stroke+DNase-I-treated mice (*n* = 5 PP per group, **P = 0.0027). **c**, 3D images showing GP1bβ^+^ platelet aggregates in CD31^+^ vasculature of PP used for analyzing surface volumes. Scale bar, 20 µm. **d**, Quantification of total GP1bβ^+^ platelet volumes to CD31^+^ surface volumes in PP isolated from sham, stroke+vehicle and stroke+DNase-I-treated mice (*n* = 5 PP per group, *P = 0.0037). Data are represented as mean ± s.d.; statistical analyses were performed by Kruskal–Wallis test.

To investigate further how circulating NETs might lead to lymphocyte death in the PP, we focused on the fact that NETs interact intensively with activated platelets in the processes of vascular thrombus formation^27^. Thus, we examined the possibility of NET-induced platelet aggregation in the PP vasculature, which may result in oxygen/nutrition deprivation and, thereby, impact lymphocyte survival in PP. Indeed, ultramicroscopic analyses of the PP vasculature showed considerably higher numbers and volumes of GP1bβ-stained platelet thrombi in stroke mice compared to sham or DNase-I-treated mice (Fig. 6a–d and Supplementary Video 3), hence suggesting that NET-induced microthrombosis might cause PP lymphocyte energy depletion.

**Extended Data Fig. 7:**
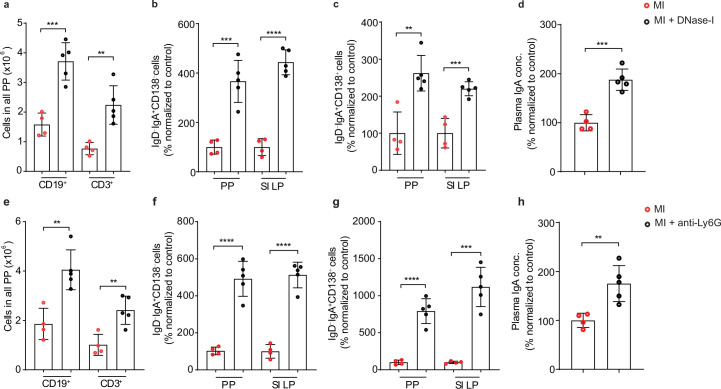
Neutrophils after MI release NETs and induce loss of plasma cells and IgA. **a**, Numbers of CD19^+^ and CD3^+^ cells in all PP in MI + vehicle and MI + DNase-I treated mice (n = 4−5 per group, unpaired *t*-test, **P = 0.0006, **P = 0.0035). **b**, Numbers of IgD^−^IgA^+^ CD138^−^ IgA-switched B cells in all PP and SI LP in MI and MI + DNase-I treated mice (n = 4−5 mice per group, unpaired t-test ***P = 0.0006, ****P < 0.0001). **c**, Numbers of IgD^−^IgA^+^ CD138^+^ plasma cells in all PP and SI LP in MI and MI + DNase-I treated mice (n = 4−5 mice per group, unpaired t-test, **P = 0.0024, ***P = 0.0005). **d**, Relative concentrations of plasma IgA in MI and MI + DNase-I treated mice (n = 4−5 per group, unpaired *t*-test ***P = 0.0003). **e**, Numbers of CD19^+^ and CD3^+^ cells in all PP in MI + isotype antibody and MI + anti-Ly6G treated mice (n = 4−6 per group, unpaired t-test, CD19^+^ **P = 0.0031, CD3^+^ **P = 0.0047). **f**, Numbers of IgD^−^IgA^+^CD138^−^ B cells in all PP and SI LP in MI + isotype antibody and MI + anti-Ly6G treated mice (n = 4−5 mice per group, unpaired t-test, ****P < 0.0001). **g**, Numbers of IgD^−^IgA^+^ CD138^+^ plasma cells in all PP and SI LP in MI + isotype antibody and MI + anti-Ly6G treated mice (n = 5−6 mice per group, unpaired t-test, ****P < 0.0001, ***P = 0.0006). **h**, Relative concentrations of plasma IgA in MI + isotype antibody and MI + anti-Ly6G treated mice (n = 4−5 per group, unpaired *t*-test **P = 0.0065). For B-D and F-H, data are presented as a percentage change and normalized to the mean of MI controls. Data represent mean ±s.d. All data is combined from at least two independent experiments, PP=Peyer’s patches, SI LP= small intestine lamina propria.

**Extended Data Fig. 8:**
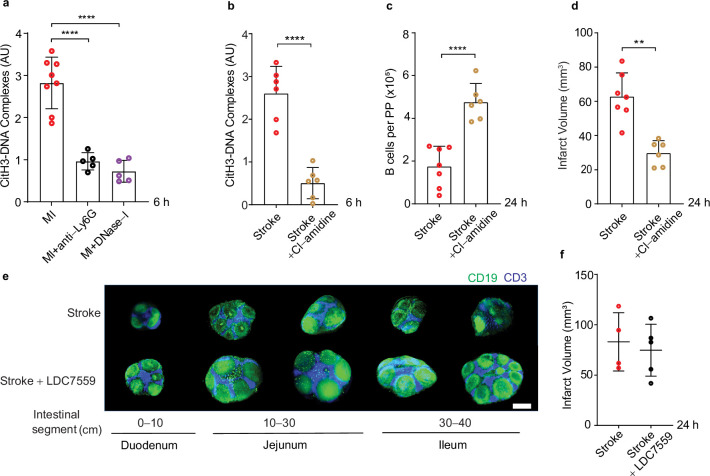
Pharmacological inhibition of NET formation blocks stroke-induced lymphocyte loss and reduction of B cell follicles in PP. **a**, Relative plasma levels of citH3−DNA complexes after 6 h of MI or MI + anti-Ly6G or MI+ DNase−I treated mice (n = 5−8 mice per group, ordinary one-way ANOVA, ****P < 0.0001). **b**, Relative plasma levels of citH3−DNA complexes after 6 h of stroke or Bi + Cl−amidine treated mice (n = 6 per group, unpaired t-test, ****P < 0.0001). **c**, Total numbers of B cells in PP of stroke + vehicle or stroke + Cl−amidine treated mice after 24 h (n = 6−7 mice per group, unpaired t-test, ****P < 0.0001). **d**, Brain infarct volumes after 24 h of stroke + vehicle or stroke + Cl−amidine treated mice (n = 6−7 per group, two−tailed Mann−Whitney U test, **P = 0.0012)**. e**, 3D reconstruction images of CD19^+^ B cells and CD3^+^ T cells in PP isolated from duodenum, jejunum and ileum 24 h of stroke + vehicle or stroke + LDC7559 treated mice, scale bar=500 µm. **f**, Brain infarct volumes after 24 h of stroke + vehicle or stroke + LDC7559 treated mice (n = 4−5 per group, two−tailed Mann−Whitney U test, *P* = 0.5556). Data represented as mean ±s.d. PP=Peyer’s patches.

Next, we tested whether NET release mediates the loss of B cells and IgA also after MI. Our results showed that NET degradation immediately after MI or depletion of neutrophils before MI reduced the loss of total CD19^+^ B cells and CD3^+^ T cells in PP (Extended Data Fig. 7a,e). Similarly, higher numbers of IgA^+^-switched B cells (Extended Data Fig. 7b,f) and IgA^+^ mature plasma cells were noted in treated MI mice (Extended Data Fig. 7c,g). Moreover, the degradation of NETs or neutrophil depletion during MI maintained increased amounts of circulating IgA (Extended Data Fig. 7d,h) and was characterized by decreased levels of NETs (Extended Data Fig. 8a).

**Fig. 7:**
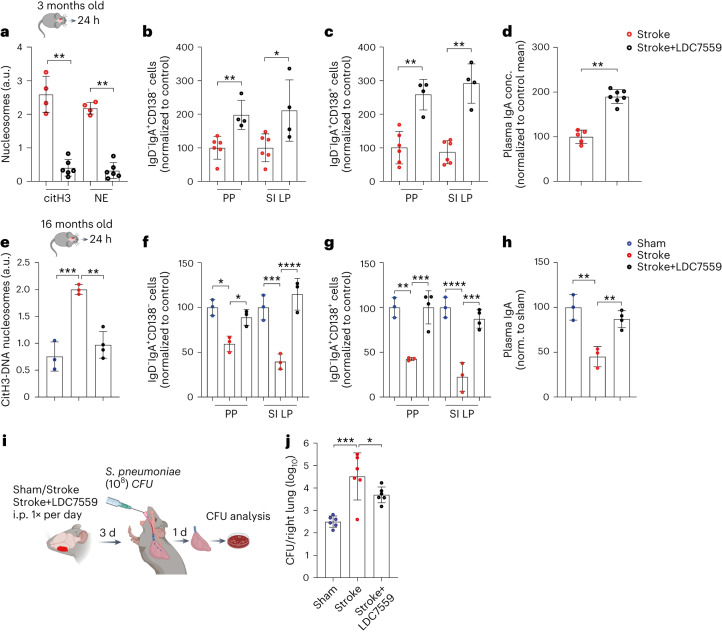
Inhibition of NETs protects IgA-secreting plasma cells after stroke in young and old mice. **a**, Relative plasma levels of citH3-DNA and NE-DNA complexes in stroke+vehicle and stroke+LDC7559-treated 3-month-old young mice (*n* = 4−6 per group, citH3 stroke versus stroke+LDC7559 **P = 0.0095, NE stroke versus stroke+LDC7559 **P = 0.0095). **b**, Numbers of IgD^−^IgA^+^CD138^−^ IgA-switched B cells in all PP and SI LP in stroke and stroke+LDC7559-treated young mice (*n* = 4−6 per group, **P = 0.0095, *P = 0.019). **c**, Numbers of IgA^+^IgD^−^CD138^+^ plasma cells in all PP and LP in stroke and stroke+LDC7559-treated young mice (n = 4−6 per group, PP comparison **P = 0.0095, SI LP comparison **P = 0.0095). **d**, Relative concentrations of plasma IgA in stroke and stroke+LDC7559-treated young mice (n = 4−7 per group, **P = 0.0025). **e**, Relative plasma levels of citH3-DNA complexes in sham, stroke+vehicle and stroke+LDC7559-treated 16-month-old mice (*n* = 3−4 per group, ordinary one-way ANOVA, ***P = 0.0007, **P = 0.0016). **f**, Numbers of IgD^−^IgA^+^CD138^−^ B cells in all PP and LP in sham, stroke and stroke+LDC7559-treated old mice (*n* = 3 per group, ordinary one-way ANOVA, PP sham versus stroke *P = 0.0165, stroke versus stroke+LDC7559 *P = 0.0144, SI LP sham versus stroke ***P = 0.0006, stroke versus stroke+LDC7559 ****P < 0.0001). **g**, Numbers of IgD^−^IgA^+^CD138^+^ plasma cells in all PP and LP in stroke and stroke+LDC7559-treated old mice (*n* = 3−4 per group, ordinary one-way ANOVA, PP sham versus stroke **P = 0.0014, stroke versus stroke+LDC7559 ***P = 0.0007, SI LP sham versus stroke ****P < 0.0001, stroke versus stroke+LDC7559 ***P = 0.0002). **h**, Relative concentrations of plasma IgA in stroke and stroke+LDC7559-treated old mice (*n* = 3−4 per group, ordinary one-way ANOVA, sham versus stroke **P = 0.0019, stroke versus stroke+LDC7559 **P = 0.0061). **i**, sham or stroke mice were treated with vehicle or LDC7559 every day, and, after 3 d, all mice were intratracheally inoculated with *S. pneumoniae* (10^8^ CFUs). Mice were euthanized 1 d after infection to analyze bacterial burden in the lungs. **j**, CFUs in the lungs of infected sham+vehicle, stroke+vehicle and stroke LDC7559 mice (*n* = 6 per group, ordinary one-way ANOVA, ***P = 0.0003, *P = 0.0195). Data represent mean ± s.d., two-tailed Mann−Whitney *U*-test (**a**–**d**). All data were combined from at least two independent experiments.

We next aimed to test whether therapeutic targeting of NET formation could maintain mucosal immune homeostasis during sterile tissue injury. To achieve this, stroke mice were first treated with the pan-peptidylarginine deiminase (PAD) inhibitor Cl-amidine, which has previously been shown to inhibit NET formation^28^. Indeed, Cl-amidine treatment reduced plasma levels of citH3-DNA complexes and prevented the loss of B cells in PP compared to vehicle-treated animals (Extended Data Fig. 8b,c). However, Cl-amidine also strongly reduced brain infarct sizes, which indicated an additional neuroprotective effect of the compound (Extended Data Fig. 8d). This, however, made it difficult to dissect the impact of NETs or reduced brain damage on B cell loss in PP. Therefore, we tested the gasdermin D inhibitor (GSDMDi) LDC7559 to block NET formation after stroke^29^. Interestingly, stroke mice treated with LDC7559 showed reduced plasma levels of citH3 and NE nucleosomes compared to the vehicle-treated group (Fig. 7a). Furthermore, the inhibition of NET formation maintained the levels of IgA-switched B cells (Fig. 7b) and mature plasma cells in PP and SI LP (Fig. 7c). In line with the increased numbers of plasma cells, we also found normalized levels of plasma IgA (Fig. 7d) and reduced shrinkage of B cell follicles in NET-inhibitor-treated stroke mice (Extended Data Fig. 8e). However, infarct volumes remained unchanged between treated and untreated groups (Extended Data Fig. 8f), hence allowing us to relate the above findings solely to the peripheral impact of NETs on lymphocyte homeostasis.

**Extended Data Fig. 9:**
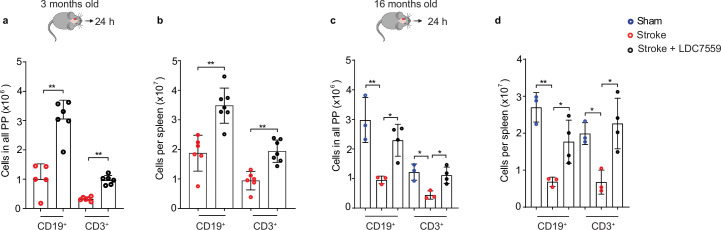
Inhibition of NETs release after stroke prevented lymphocyte loss in PP and spleen in young and old mice. **a**, Numbers of CD19^+^ B cells and CD3^+^ T cells in PP of sham, stroke and stroke + LDC7559 treated young mice (n = 5−6 mice per group, two−tailed Mann−Whitney U test, CD19^+^ **P = 0.0043, CD3^+^ **P = 0.0022). **b**, Numbers of CD19^+^ B cells and CD3^+^ T cells in spleens of sham, stroke and stroke + LDC7559 treated young mice (n = 6−7 mice per group, two−tailed Mann−Whitney U test, **P = 0.0012). **c**, Numbers of CD19^+^ B cells and CD3^+^ T cells in PP of sham, stroke and stroke + LDC7559 treated old aged mice (n = 3−4 mice per group, ordinary one-way ANOVA, CD19^+^ sham vs stroke **P = 0.0078, stroke vs stroke+LDC7559 *P = 0.0427, CD3^+^ sham vs stroke *P = 0.0183, stroke vs stroke+LDC7559 *P = 0.027). **d**, Numbers of CD19^+^ B cells and CD3^+^ T cells in spleens of sham, stroke and stroke + LDC7559 treated aged mice (n = 3−4 mice per group, ordinary one-way ANOVA, CD19^+^ sham vs stroke **P = 0.0025, stroke vs stroke+LDC7559 *P = 0.0438, CD3^+^ sham vs stroke *P = 0.0463, stroke vs stroke+LDC7559 *P = 0.0138). Data represent mean ±s.d., All data is combined from at least two independent experiments, PP=Peyer’s patches.

Most strokes occur in elderly patients (>65 years of age) and are often associated with concomitant bacterial infections, causing poor disease outcomes^30^. To test whether NET formation after stroke impacts intestinal B cell survival in aged animals, we performed experiments in 16-month-old mice. We observed a similar induction of citH3-DNA complexes, loss of IgA-switched B cells and mature plasma cells in PP and SI LP after stroke in aged mice as compared to young mice (Fig. 7e–g). Moreover, stroke in aged mice also reduced the amounts of circulating IgA (Fig. 7h). The findings that the GSDMDi reduced the loss of total B and T cells in PP and spleen after stroke in both young and aged mice further support the significance of this pathway in systemic and intestinal lymphoid tissues (Extended Data Fig. 9a–d). Notably, these mechanisms appear independent of the host age and are influenced only by the event of ischemic stroke-mediated brain injury.

Immunodeficiency after sterile inflammation often makes patients susceptible to bacterial respiratory tract infections^11^. Therefore, we investigated whether inhibition of NETs, which prevents the loss of lymphocytes (Fig. 7b,f) and stabilizes plasma IgA (Fig. 7d,h) after stroke, can reduce lung bacterial burden in infected mice. For this, stroke mice were treated with LDC7559 for 3 d after injury and then infected with *Streptococcus pneumoniae* and analyzed for bacterial load in the lungs 1 d later (Fig. 7i). Strikingly, stroke mice showed more than 100-fold increased bacterial counts in the lungs compared to infected sham mice. This high burden was reduced more than six-fold by the inhibition of NET release after brain injury (Fig. 7j).

**Fig. 8:**
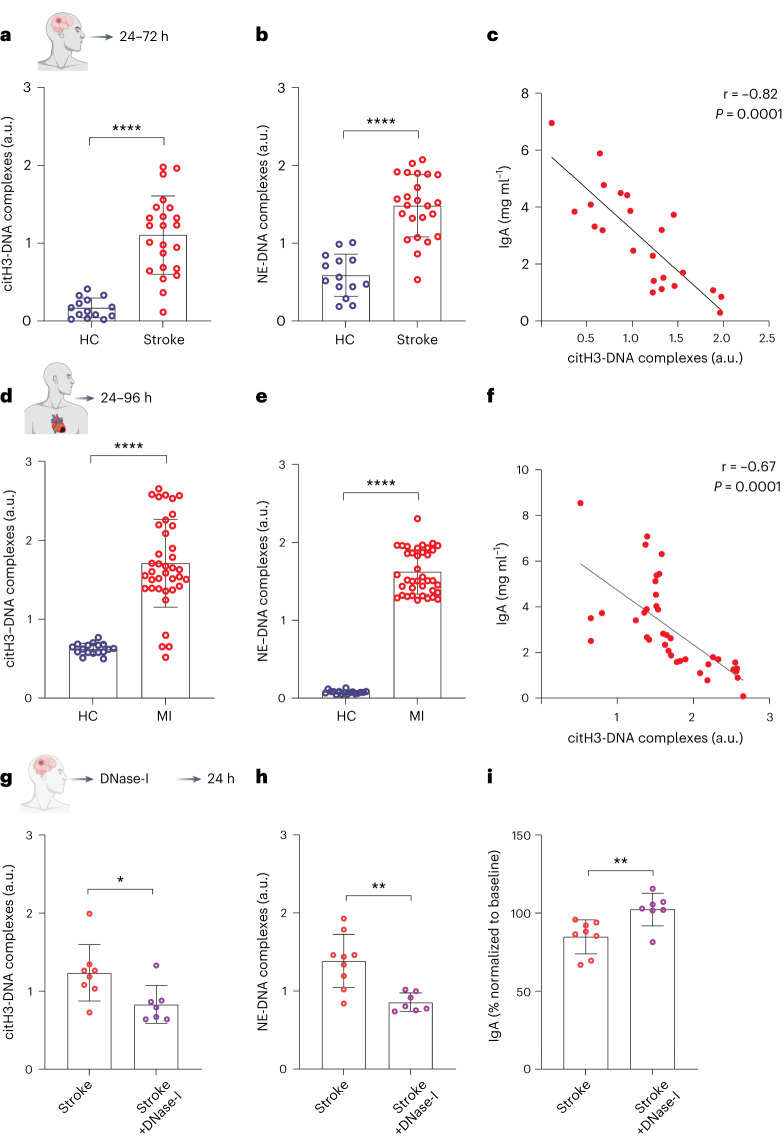
Patients with stroke and MI show increased circulating NETs and reduced IgA levels that can be treated with DNase-I. **a**,**b**, Relative plasma levels of citH3−DNA (**a**) and NE−DNA complexes (**b**) in patients with stroke and healthy individuals (*n* = 14−23 per group, ****P < 0.0001). **c**, A signifi**c**ant negative correlation between plasma citH3-DNA complex levels and IgA amounts in patients with stroke. **d**,**e**, Relative plasma levels of citH3-DNA (**d**) and NE-DNA complexes in patients with MI and healthy controls (*n* = 17−38 per group, ****P < 0.0001). **f**, A significant negative correlation between plasma citH3-DNA complex levels and IgA amounts in MI (*n* = 17−38 per group). **g**,**h**, citH3-DNA (*n* = 7−8 per group, *P = 0.0263) (**g**) or NE−DNA (*n* = 7−9 per group, **P = 0.0012) (**h**) complexes at 24 h after therapy in the plasma of patients with stroke treated at timepoint 0 with tPA alone or combined with recombinant human DNase-I. **i**, Levels of plasma IgA in the same patients with stroke (*n* = 7−8 per group, **P = 0.0093). The IgA data were normalized to the baseline plasma IgA levels of the same individuals before the administration of treatment, and percentage values are presented. Data represent mean ± s.d., Mann−Whitney *U*-test. HC, healthy control; MI, myocardial infarction.

To evaluate the relevance of our experimental findings for the clinical reality, we analyzed circulating NETs in plasma samples of humans with ischemic stroke and MI. Our data showed higher plasma levels of citH3-DNA and NE-DNA complexes in patients with stroke compared to the healthy individuals (Fig. 8a,b). Strikingly, the reduced levels of plasma IgA in patients with stroke were highly significantly correlated with increasing amounts of circulating citH3-DNA complexes (Fig. 8c). Furthermore, the plasma levels of citH3-DNA and NE-DNA complexes were also increased in patients with MI compared to healthy individuals (Fig. 8d,e), and also, here, plasma IgA levels were negatively associated with increased plasma amounts of NETs (Fig. 8f).

Finally, to confirm the causality of NET-induced IgA loss after stroke in humans, we analyzed the plasma samples (before and 24 h after treatment) of patients treated with standard thrombolysis plus recombinant human DNase-I compared to only thrombolyzed patients adjusted for age and baseline stroke severity in a small-scale clinical trial (https://classic.clinicaltrials.gov/show/NCT04785066). Interestingly, DNase-I therapy reduced the amounts of circulating NETs (Fig. 8g,h). This was associated with stabilization of 24-h plasma IgA levels compared to patients under standard therapy who experienced a substantial IgA drop on the first day after stroke diagnosis (Fig. 8i).

Altogether, our results highlight a previously unknown mechanism of NET-induced mucosal B and T cell death causing immune dysfunction after sterile tissue injuries.

## Discussion

In the present study, we found that both stroke and MI trigger extensive lymphocyte death in the intestinal mucosal tissues, leading to a reduction in systemic Ig levels. Interestingly, lymphocyte death was mediated via NETs released from activated neutrophils after tissue injury.

The lower antibody concentrations in patients with stroke may increase susceptibility to bacterial infections^10^. Of note, more than 70% of detected bacteria in infected patients belong to human intestinal commensals, suggesting the translocation of bacteria through leaky intestinal mucosal barriers^3^. Consequently, mature IgA-secreting plasma cells are key effectors, because they protect mucosal barriers via IgA secretion. The secretory forms of IgA have a short half-life in the timeframe of hours^6^, and immune defenses are supplemented with IgG whose half-life in circulation is many days^7^. This study revealed that the events leading to lymphocyte death after sterile inflammation are not limited to specific cellular subtypes, such as IgA-producing cells, but, in contrast, appear to affect all lymphocytes equally. Of note, SI LP preparations include isolated lymphoid follicles (ILFs), small lymphoid tissues made up mainly of resting naive B cells.

It is clear from our data that both types of tissue injury affect not only B cells but also T cells in studied intestinal tissues. T cells play a major role in the development of Ig-producing B cells in lymphoid tissues, and their loss can also indirectly modulate B cell functions. A specific subset of B cells can synthesize neurotrophins to support neuronal survival^31^. B cell function in neuroregeneration after stroke^32^ might be essential to counteract neuroinflammation induced by intestinal inflammatory T cells^33^. The observed long-term loss of B cells in PP after stroke might have worse consequences on intestinal inflammation and long-term brain recovery. The induction of early B cell loss and ongoing metabolic distress in the remaining cells in PP is likely to cause the reduction in circulating IgA to a large extent. Further effects are probably caused by an active loss of plasma cells in intestinal tissues and a massive loss of splenic B cells.

Previous studies showed ciDNA as an inducer of splenic T cell death^15^. The source of the surplus ciDNA underlying B cell loss after sterile injury, which was so far elusive, was identified in our study to be hyperactivated neutrophils. Several soluble mediators released after sterile tissue injury have been discussed to play a role in activating neutrophils that then induce the formation of toxic NETs—for example, platelet-released HMGB1 (ref. ^34^). NETs consist of DNA, histones and granular proteins and increase collateral inflammation and cell death. A recent study showed that a synergy between histones and DNA in NETs is required to trigger inflammation and cellular toxicity^22^. Our data that DNase-I treatment inhibits lymphocyte loss in PP after tissue injury and can completely remove microvascular thrombi suggest a similar loss of function of NETs after disruption of the DNA backbone.

Recently, we showed that NETs directly induce smooth muscle cell death in arterial inflammation via attached histone H4 proteins^24^. The fact that treatment of B cells with stroke plasma increases apoptotic B cells also shows direct toxicity of NETs or its components. However, this study could not detect NETs in the proximity of lymphocytes in PP, and, hence, it remains elusive whether direct molecular interactions of NET components induce lymphocyte death in PP. NETs play a key role in the formation of vascular thrombosis during sterile tissue injury^27^. The observed extensive thrombus formation in the vasculature of PP after stroke suggests the attractive possibility that the compromised local blood supply affects cell metabolism and, thereby, induces cell death. Defective PP perfusion may also explain the observed long-lasting loss of B cells in PP after stroke.

In addition to NET-mediated loss of immune cells, possibly other factors may induce these changes, such as the intestinal microbiome^35^. However, our findings strongly support the contribution of NETs in triggering B cell loss in intestinal tissues after stroke and MI. Recent studies have suggested GSDMD functions in mediating the release of active IL-1β and NETs by activated neutrophils in mouse models of tissue injury^29,36^. Moreover, NET formation is substantially higher in the elderly, and patients with increased age often present higher infection rates and mortality after stroke^37^. Inhibition of GSDMD with LDC7559 in our study blocked the release of NETs and loss of plasma cells in young and aged mice. Our demonstration that this also directly impacts the bacterial burden in the lungs of stroke mice makes GSDMD a prime target for innovative immunoprotective treatments in sterile inflammation.

Previous studies showed the detrimental effects of NETs on stroke outcome^34^. Our study shows higher levels of circulating NETs in patients with stroke and MI that correlate with reduced IgA levels. Notably, our clinical results with DNase-I treatment fully confirm the experimental data showing the negative impact of NETs on intestinal B cell survival and the amounts of circulating IgA. At present, three registered clinical trials worldwide, including our own, are testing the safety and efficacy of recombinant human DNase-I treatment in patients with ischemic stroke (https://classic.clinicaltrials.gov/show/NCT04785066, https://classic.clinicaltrials.gov/show/NCT05880524 and https://classic.clinicaltrials.gov/show/NCT05203224). The primary endpoints of these clinical trials are to analyze improved early reperfusion and neurological outcomes, but our findings now also show that early NET degradation in patients with stroke might be an appropriate treatment to improve IgA levels. These data suggest that the focus of ongoing clinical stroke trials should be expanded from evaluating neuroprotective outcomes alone to including immunoprotective effects, which could also have beneficial consequences for patients. With our identification of GSDMD as a target for specifically blocking NET release, further trials addressing this pathway are conceivable.

Collectively, our findings demonstrate that stroke and MI lead to a substantial reduction in the amounts of circulating Ig via the induction of NET release and B cell death, especially in intestinal mucosal tissues. These results open attractive treatment options by targeting NET release or function—for example, via GSDMD blockade—as emergency therapy in patients with stroke or MI.

## Methods

### Mice

All animal experiments were performed following ethical guidelines and were approved by the local authority, Landesamt für Natur, Umwelt und Verbraucherschutz, under permission numbers G1713/18, G1719/19, G1650/17 and G1757/19. In all experiments, male C57BL/6JHsd wild-type mice (10–12 weeks old) were received from Envigo, Netherlands, for stroke experiments, and male C57BL/6JRj wild-type male mice (10–12 weeks old) were obtained from Janvier Labs, France, for MI experiments. For experiments on aged mice, 16-month-old C57BL/6JHsd wild-type male mice were used. Mice were housed in individually ventilated cages with dark/night cycles (12 h/12 h). The room temperature was 21–23 °C with 40–60% humidity. The intestinal tissues of 10-week-old male *Igh-J*^*tm1Cgn*^/J (J_H_T^−/−^) mice were received from Ari Waisman. Mice were randomly divided into two groups that underwent sham surgery or ischemic brain or myocardial injury. All experiments were performed and reported according to ARRIVE guidelines.

### Mouse model of brain injury

Brain injury was induced by tMCAO. In brief, a laser Doppler flow probe was attached to the skull above the core of the middle cerebral artery (MCA) territory. Mice were placed on a feedback-controlled heat pad during surgery. The common carotid artery (CCA) and left external carotid arteries were identified and ligated. A 2-mm silicon-coated filament (702234PK5Re, Doccol) was inserted into the internal carotid artery to occlude the MCA. Brain ischemia was validated by a stable reduction of blood flow to ≤20% of baseline. After 1 h of occlusion, the filament was removed for the reestablishment of blood flow. Preoperative buprenorphine (0.1 mg kg^−1^ body weight, subcutaneous (s.c.)) and postoperative carprofen (4–5 mg kg^−1^ body weight, s.c.) were injected. Sham-operated mice underwent the same surgical protocol except that the filament was inserted into the CCA and immediately removed.

### Mouse model of myocardial injury

Male C57BL/6JRJ mice (aged 9–15 weeks) were subjected to a myocardial ischemia–reperfusion injury. In brief, mice were anesthetized with ketamine (100 mg kg^−1^, intraperitoneal (i.p.)) and xylazine (10 mg kg^−1^). Mice were then orally intubated and ventilated throughout the operation procedure, with 0.8 L min^−1^ air and 0.2 L min^−1^ O_2_ at a tidal volume of 250 μl per stroke and a breathing frequency of 140 strokes per mininute with 2% isoflurane to ventilated air as maintenance anesthesia. After the left lateral thoracotomy, the left coronary artery was ligated. After 45 min of ischemia, the affected myocardium was reperfused.

### Clinical patient populations

All study participants or legal surrogates provided written informed consent following the Declaration of Helsinki and the International Conference on Harmonization Guidelines for Good Clinical Practice. All regulations were followed according to local authorities and according to the clinical protocols. No compensation was offered for study participation. Patient cohort details can be found in Supplementary Table 1. Patients with ischemic stroke were recruited within 10 d of symptom onset through the emergency department at the University Hospital Essen, Germany. All patients had a first-time diagnosis of ischemic stroke, and their stroke severity was defined according to the National Institutes of Health Stroke Scale. Ethical approval for use of the plama of healthy individuals and patients with stroke was granted as per the institutional ethics board committee of the University Hospital Essen (study numbers 18-8408-BO and 23-11200-BO).

Human plasma samples from acute ischemic stroke patients treated with or without DNase-I were collected from the NeutroStroke and IMPRESS studies (ClinicalTrials.gov: NCT02900833 and NCT04663399; https://classic.clinicaltrials.gov/show/NCT04785066) at the Rothschild Foundation Hospital, Paris, France, with the stated approval numbers. Blood was collected in EDTA (BD Vacutainer 4 ml, K3E 7.2 mg) from the femoral artery for the baseline (before endovascular therapy (EVT)) and from a venous brachial puncture at 24 h after EVT. Ethical approval for use of the plasma of healthy controls and patients with MI was granted as per the institutional ethics board committee of the University Hospital Essen (study number 23-11200-BO) and from the Universität Münster, Münster, Germany (study numbers 2021-424-f-S and 2021-532-f-S). Blood samples were taken within 4 d after ST-segment elevation MI.

### Quantification of brain infarct volumes

For the calculation of brain infarct volumes, brains were removed and frozen on dry ice. Then, 20-μm cryosections were cut at 500-μm intervals and stained with cresyl violet. All sections were scanned using 600 dpi and analyzed using ImageJ software (National Institutes of Health). Using a scale of 23.62 pixels per millimeter, the area of non-stained infarct tissue was measured and integrated into the total brain. An edema correction for brain infarct volume was performed using the following formula: (ischemic area) = (direct lesion volume) − [(ipsilateral hemisphere) − (contralateral hemisphere)]. The lesion volume per hemisphere was presented as mm^3^.

### Single-cell preparations from lymphoid organs and flow cytometry analysis

Mice were deeply anesthetized, and blood was collected via cardiac puncture and added into EDTA-containing tubes. Mice were perfused with PBS, and the spleen, BM, mLN and PP were collected in cold PBS. Single-cell suspensions were prepared by mincing the tissues in PBS and filtered through 70-μm cell filters. Single-cell suspensions were kept on ice until further use. After the isolation of PP, LP lymphocytes were isolated^38^. In brief, small intestine was washed and transferred into 15 ml of EDTA-DTT buffer containing 1 mM EDTA and 1 mM DTT and incubated at 37 °C and 250 r.p.m. for 15 min on a shaker. Afterwards, samples were filtered through a 70-µm filter, and the remaining tissues were transferred to digestion media containing 5 µg ml^−1^ Liberase (5401054001, Roche) and 10 µg ml^−1^ DNase-I (11284932001, Roche), incubated at 37 °C and 250 r.p.m. for 35 min. Samples were filtered through a 70-µm cell strainer and washed with PBS. After centrifugation at 450*g* for 10 min, cell pellets were resuspended in 44% Percoll and layered over 67% Percoll solution. Samples were then centrifuged at 520*g* for 30 min at 14 °C. Cells at the interface were collected, counted and used for staining with fluorochrome-conjugated antibodies against cell surface markers. Detailed information on used antibodies and reagents is provided in Supplementary Tables 2 and 3. Stained cells were measured on a BD FACSAria flow cytometer and analyzed using FlowJo software.

### PP cell culture and plasma treatment

The single-cell preparations were prepared from PP isolated from naive mice and plated into 96-well plates at a density of 2.5 × 10^5^ per well in TexMACS Medium (130-097-196, Miltenyi Biotec) with 1% penicillin–streptomycin. Mice were euthanized 6 h after surgery, and plasma was prepared by centrifugation of EDTA-blood at 8,000*g* for 10 min. Afterwards, cells were treated with plasma from stroke or sham-operated mice at 30% final concentration. For the degradation of DNA in stroke plasma, samples were treated with DNase-I (10 U ml^−1^) at 37 °C for 30 min and then added to the cell cultures. Cells were incubated at 37 °C in 5% CO_2_ for 12 h. At the end of incubation, cell death stainings were performed.

### Cell death stainings

Caspase-3/7 reagent (C10427, Thermo Fisher Scientific) was added to the cell cultures and incubated at 37 °C in 5% CO_2_ for 30 min. In addition, apoptotic cells were detected using Annexin V (640906, BioLegend) and propidium iodide (P4864, Sigma-Aldrich) staining followed by surface staining with cell surface markers and flow cytometry analysis.

### In vivo degradation of ciDNA and inhibition of NET release

For the degradation of ciDNA, mice were injected immediately after the onset of stroke or MI with recombinant DNase-I (11284932001, Roche; 1,000 U per mouse in 100 µl of saline, intravenous (i.v.)). NET generation was inhibited using Cl-amidine or LDC7559. The stock solution of Cl-amidine (506282, Millipore) was dissolved in dimethyl sulfoxide and then further diluted in PBS. After brain injury induction, Cl-amidine was injected i.p. at 10 mg kg^−1^. The stock solution of LDC7559 (HY−111674, MedChemExpress) was dissolved in DMSO and then further diluted in corn oil and injected i.p. at 10 mg kg^−1^ after surgery. Control mice were injected with an equal amount of vehicles.

### Depletion of neutrophils and B cells

Neutrophil depletion was achieved by the injection of anti-Ly6G antibody (BE0075-25, Bio X Cell, 100 µg per mouse, i.p.) followed by anti-rat antibody (BE0122, 100 µg per mouse, i.p.) injection on the next day. Mice were once more injected with anti-Ly6G antibody (100 µg per mouse) before performing surgery. Control mice received the same amounts of rat IgG2a isotype antibodies (BE0089, Bio X Cell). B cells in naive mice were depleted by the injection of anti-CD20 antibody (BE0356, Bio X Cell, 100 µg per mouse, i.p.). The depletion of circulating neutrophils was verified by intracellular staining with fluorochrome-conjugated Ly6G antibody (clone 1A/8) using a FoxP3/transcription factor staining kit followed by flow cytometry analysis.

### Optical clearing of PP

The intestinal tissues containing PP were embedded in 1.5% low-melting agarose and fixed with 4% paraformaldehyde (PFA) in PBS overnight at 4 °C. Samples were then treated with a series of ethanol (EtOH) solutions starting from 30% and 60%, 80% and two times 100% for 12 h on an orbital shaker (50 r.p.m. at 4 °C). At last, samples were treated with ethyl cinnamate (ECi) to match the refractive index, and cleared samples were imaged using LSFM (UltraMicroscope Blaze, Miltenyi Biotec). The images were acquired with a zoom factor of ×3.2 with an interval of 5 µm. For volume analysis, the images were processed using Imaris software version 9.5.1.

### Whole-mount staining of PP and LSFM analysis

Intestinal samples were fixed in 4% PFA and transferred to 1 ml of permeabilization buffer containing 20% DMSO, 1% Triton X-100 and 2.3 g per 100 ml of glycine in PBS. Samples were treated with a blocking buffer containing 3% rat serum and anti-mouse CD16/32 antibody for 30 min. Then, samples were incubated with anti-CD19 AF594 and anti-CD3 AF647 antibodies overnight. In separate experiments requiring microvascular staining, mice were injected with anti-CD31 and anti-GPIbβ antibodies and euthanized after 30 min to prepare intestinal tissues. Samples were washed and embedded in 1.5% low-gelling agarose and dehydrated in serial dilutions of EtOH (40%, 60%, 80% and 100%) for 1 h in each solution. Samples were finally transferred to ECi, and cleared samples were imaged by LSFM. The quantification of GPIbβ^+^ volumes as an indicator of platelet aggregation and total GPIbβ^+^ thrombi volume in CD31^+^ blood vessels was performed using the Imaris surface rendering function.

### Quantification of IgA and IgG

Patient plasma was collected after centrifugation of EDTA blood and used to measure Ig levels using ELISA kits, as described by the manufacturer (IgA, 88-50600-22, Thermo Fisher Scientific; IgG, 88-50550-88, Thermo Fisher Scientific). Mice plasma and fecal samples were processed and used to measure Ig levels using immunoassay kits as described by the manufacturer (IgA, 88-50600-22, Thermo Fisher Scientific; IgG, 88-50550-88, Thermo Fisher Scientific).

### Quantification of plasma DNA and NETs in plasma samples

Total DNA amounts in plasma were measured using a Qubit dsDNA HS Assay Kit (Q32851, Thermo Fisher Scientific). NET quantification was performed based on citH3 or NE associated with DNA^39,40^. Anti-histone H3 antibody or NE antibody coated 96-well plates were prepared to measure NET amounts using a Cell Death ELISA^PLUS^ Kit (11774425001, Roche).

### Purification of B cells for RNA-seq and liquid chromatography–mass spectrometry

B cells from intestinal PP were purified using a Pan B Cell Isolation Kit II as described by the manufacturer (130-095-813, Miltenyi Biotec), providing B cells with small amounts of intestinal epithelial and endothelial cells. To further enrich B cell preparations, cell suspensions were further purified using direct B cell labeling with CD45 microbeads using MojoSort Mouse CD45 Nanobeads (480028, BioLegend). This isolation procedure provides B cells with a purity of 99% or greater. Afterwards, RNA was isolated using an RNeasy Micro Kit (74004, Qiagen).

### RNA-seq analysis

The quality of sequenced reads was determined with FastQC (version 0.11.9)^41^ and Illumina adapters, and low-quality reads with Phred scores less than 20 were trimmed with Trimmomatic (version 0.39)^42^. Kallisto (version 0.48.0-1)^41^ was used to pseudo-align reads to the GRCm38 release 102 genome assembly from Ensembl and subsequently to quantify transcript expression as transcripts per million (TPM). Transcripts with less than 1 TPM across all 12 samples were removed, and, in R (version 4.1.2), a Wald test from the Sleuth package (version 0.30.0)^43^ was used to analyze differential expression between the sham-operated and stroke sample groups at the gene level, with significance set at *P* < 0.05. Gene Ontology (GO) Biological Process^44^ enrichment analysis was also performed in R, with the clusterprofiler package (version 4.0.5)^45,46^, and significance was defined as FDR-adjusted *P* < 0.05. In GSEA, the dot size indicates the ratio of the overlap of differentially expressed genes with single GO term gene sets to the overlap of differentially expressed genes with the sum of all unique GO term genes queried.

### Cryosectioning and staining of PP

Intestinal PP were fixed frozen, and 10-µm-thick histological sections were prepared. The sections were stained with antibodies CD3-AF647 (100209, BioLegend) CD19-AF594 (115552, BioLegend) and anti-MPO (ab9535, Abcam) in a blocking buffer at 4 °C overnight. For a different set of staining, intestinal sections were stained with EpCAM-AF594 (118222, BioLegend) and GL7-AF647 (144606, BioLegend) in a blocking buffer at 4 °C overnight. For MPO staining, sections were washed three times in DPBS and incubated with secondary antibody donkey anti-rabbit AF488 (A32790, Invitrogen) in a blocking buffer for 2 h at room temperature. All sections were then washed three times with DPBS, and cell nuclei were stained with DAPI for 10 min at room temperature. Sections were washed and mounted in Dako Fluorescence Mounting Medium (Agilent Technologies) and analyzed using a confocal microscope.

### Proteomics analysis of circulating neutrophils and PP B cells

Purified blood neutrophils and PP B cells were used for proteomics analysis. The tryptic digest peptides were taken forward for label-free quantitative proteomic analysis on an Orbitrap Eclipse Tribid Mass Spectrometer coupled to a nanoflow liquid chromatography system. The peptides were separated on a C-18 reversed-phase nanocolumn (Acclaim PepMap 100, 75 μm × 50 cm, Thermo Fisher Scientific) with the gradient of 3–35% solvent B (B is 84% acetonitrile and 0.1% formic acid) for 90 min at a flow rate of approximately 400 nl min^−1^. The eluted peptides from the C-18 nanocolumn were subjected to nanospray ionization and analyzed by an Orbitrap mass analyzer at a resolution of 120,000, followed by high-energy collision dissociation and tandem mass spectrometry spectra analysis by ion trap. All data were acquired in a data-dependent manner with a 3-s cycle time. All mass spectrometry raw datasets were analyzed using Proteome Discoverer version 2.5.0.400 with an in-built search engine, MASCOT, against the UniProt mouse database (UP000000589, downloaded on 10 August 2019). Missing values were imputed with low sampling abundance. The data were further normalized based on the total peptide amount and scaled before statistical analysis (that is, *t*-test and FC). Functional enrichment of statistically significant proteins was performed in g:Profiler. Data visualization was done using R (https://github.com/Susmita-isas/Stroke-mice-/new/main and https://ggvolcanor.erc.monash.edu/) and SR plot.

### Intratracheal *S. pneumoniae* infection model

For bacterial lung infection, a pneumococcal serotype 1 strain (*S. pneumoniae* SV1, American Type Culture Collection, 33400) was used as described previously^47^. In brief, *S. pneumoniae* culture was grown overnight on Columbia blood agar plates (Oxoid, PB5039A) at 37 °C; single colonies were resuspended and cultured in 10 ml of Brain Heart Infusion broth (Thermo Fisher Scientific, TV5090E) to mid-logarithmic phase (OD_600_ = 0.045–0.055; NanoDrop 1000); and 800 µl of culture was frozen at −80 °C with 200 µl of 86% glycerol. For infection, bacteria were cultured to mid-logarithmic phase, centrifuged at 1,500*g* for 10 min at 4 C and resuspended in 550 µl of PBS. Then, 50 µl bacterial solution per animal corresponding to 1 × 10^8^ colony-forming units (CFUs) of *S. pneumoniae* was used for infection. Mice were intratracheally inoculated with bacteria after 3 d of sham or stroke surgery. One day after infection, mice were euthanized, and different tissues were collected. Lungs were removed aseptically, and right lung lobes were analyzed for bacterial cultures by dissociating in 2 ml of PBS via gentleMACS dissociator M tubes with a cell strainer. After centrifugation, supernatants were plated onto blood agar plates in 10-fold dilutions and incubated for 24 h at 37 °C. Afterwards, CFUs on blood agar plates were counted.

### Deep-learning-based pipeline for B cell volume analysis of 3D images

The volume of B cells and T cells was measured from the segmentation results of the corresponding channels of the 3D LSFM images. A human-in-the-loop deep-learning-based segmentation method was employed to obtain the final segmentation results. The overall segmentation workflow was generalized from the iterative deep learning workflow in the Allen Cell and Structure Segmenter and demonstrated in Extended Data Fig. 1h. The images were downsampled in *x*–*y* dimensions by a ratio of 0.25. The neural network architecture was the enhanced 3D UNet, trained with a weighted sum of DICE loss and cross-entropy loss on a single NVIDIA A100 GPU. For the final volume analysis, volumes in pixels obtained from volume analysis were converted to micrometer cubes, by multiplying with the voxel size of 0.975 × 0.975 at 5-µm intervals.

### Statistical analysis

Data were analyzed using GraphPad Prism version 9.0. After testing for normality using the Shapiro–Wilk normality test, for comparisons between more than two Gaussian distributed groups, ordinary one-way ANOVA with Bonferroni’s multiple comparison post hoc tests were used. The comparisons between the two non-Gaussian distributed groups were analyzed using the Kruskal–Wallis test. Non-Gaussian distributed two groups were compared via two-tailed Mann–Whitney *U*-test, and Gaussian distributed two groups were compared via *t*-test. Differences with *P* ≤ 0.05 were considered statistically significant.

### Reporting summary

Further information on research design is available in the Nature Portfolio Reporting Summary linked to this article.

### Supplementary information

Supplementary Tables Supplementary Table 1: Description of clinical sampling and patient information. Supplementary Table 2: List of commercial antibodies. Supplementary Table 3: List of commercial kits and chemicals.

Reporting Summary

Video 1 Cellular architecture of PP.

Video 2 PP size after sham and stroke.

Video 3 Vascular platelet aggregation in PP after stroke.

### Source data

Source Data Fig. 1 Source data for all figures and extended data figures.

## Data Availability

All data supporting the findings in this study are included in the main article and its associated files. All RNA-seq and mass spectrometry data can be found under accession numbers GSE254410 and PXD044644, respectively.

## Code availability

All details for model training and inference can be found at https://github.com/MMV-Lab/peyers_patch^48^. The trained models are available at https://zenodo.org/record/6302990#.YhyocqvMI2x for reproducibility^49^.
