# Supplementary material for: Stroke and myocardial infarction induce neutrophil extracellular trap release disrupting lymphoid organ structure and immunoglobulin secretion
Source: Nat Cardiovasc Res. 2024 Apr 23;3(5):525–40. doi: 10.1038/s44161-024-00462-8 (PMC11358010; doi:10.1038/s44161-024-00462-8)
Supplement: Supplementary file 1 — Supplementary Table 1: Description of clinical sampling and patient information. Supplementary Table 2: List of commercial antibodies. Supplementary Table 3: List of commercial kits and chemicals. [file 44161_2024_462_MOESM1_ESM.pdf]

nature cardiovascular research

Article https://doi.org/10.1038/s44161-024-00462-8

Stroke and myocardial infarction induce neutrophil extracellular trap release disrupting lymphoid organ structure and immunoglobulin secretion

In the format provided by the authors and unedited

Supplementary Table 1: Description of clinical sampling and patient information.

**Stroke patients**	**Stroke (n=23)**	**Healthy controls (n=15)**
Age, mean (SD)	68 (12)	67 (11)
Sex (female%)	21%	21%
NIHSS, mean	15.4	n/a
Sampling time after stroke (mean [SD])	1 d – 10 d (31.7 h [18 h])	n/a

**DNase-I treated stroke patients**	**Stroke (n=9)**	**Stroke + DNase-I (n=7)**
Age, mean (SD)	74.1 (8)	74.6 (11)
Sex (female%)	5/9 (56%)	6/7 (86%)
NIHSS, mean	18.25	19.5
Sampling time	0 and 24 h	0 and 24 h

**Myocardial infarction patients**	**Myocardial infarction (n=38)**	**Healthy controls (n=17)**
Age, mean (SD)	60 (12)	60 (11)
Sex (female%)	10%	11%
Sampling time after MI (mean [SD])	0-96 h (54 h [22.7])	n/a

Supplementary Table 2: List of commercial antibodies

Company	Catalog Number	Marker	Clone	Fluorophore	Dilution
BioLegend	103140	CD45	30-F11	Brilliant Violet 605	1:400
BioLegend	100204	CD3	17A2	FITC	1:200
BioLegend	127608	Ly6G	1A/8	PE	1:500
BioLegend	127608	IgA	mA-6E1		1:500
BioLegend	142506	CD138	281-2	APC	1:500
Thermo Fisher	48-5993-82	IgD	11-26C	eFluor 450	1:200
BioLegend	144604	GL7	GL7	FITC	1:100
BioLegend	103244	B220	RA3-6B2	Brilliant Violet 605	1:250
BioLegend	406001	IgG	Poly4060	FITC	1:200
Thermo Fisher	48-0112-82	CD11b	M1/70	eFluor 450	1:200
Thermo Fisher	17-5790-82	IgM	II/41	APC	1:400
BioXcell	BE0122	Mouse, anti-rat Kappa Immunoglobin Light Chain	MAR 18.5		100 µg/mouse
BioXcell	BE0356	Anti-CD20	MB20-11		100 µg/mouse
BioXcell	BE0089	Rat IgG2a isotype control	2A3		100 µg/mouse
BioXcell	BE0075	Anti-Ly6G	RB6-8C5		100 µg/mouse
BioLegend	115552	CD19	6D5	AlexaFluor 594	1:100
BioLegend	100209	CD3	17A2	AlexaFluor 647	1:100
BioLegend	102528	CD31	MEC13.3	APC/Fire 750	7.5 µg
Emfret	X-649	Gp1b-beta	Anti-gPIb beta derivative	DyLight649	3 µg
BioLegend	127626	Ly6G	1A8	AlexaFluor 488	1:200
Abcam	Ab5103	citH3	polyclonal	unconjugated	1:200
Abcam	Ab9535	MPO	polyclonal	unconjugated	1:200
BioLegend	118222	EpCAM	G8.8	AlexaFluor 594	1:100
BioLegend	144606	GL7	GL7	AlexaFluor 647	1:100
Carl Roth	2871890-3	DAPI			1:500
BioLegend	101320	CD16/32	93	TruStain FcX	1:1000
Abcam	Ab68672	Neutrophil elastase	polyconal	unconjugated	5 µg/mL
Thermo Fisher	A32790	Donkey, anti Rabbit IgG		AlexaFluor 488	1:200
BioLegend	152410	CD19	1D3	APC	1:500

Supplementary Table 3: List of commercial kits and chemicals

**Kits**	**Company**	**Catalog Number**
FoxP3/transcription Factor staining kit	Thermo Fisher Scientific	00-5523-00
ELISA kit IgA	Thermo Fisher Scientific	88-50600-22
ELISA kit IgG	Thermo Fisher Scientific	88-50550-88
Immunoassay kit IgA	Thermo Fisher Scientific	**88-0450-22**
Immunoassay kit IgG	Thermo Fisher Scientific	**88-50400-22**
Qubit™ dsDNA HS-Assay-Kit	Thermo Fisher Scientific	Q32851
Cell Death ELISA^{PLUS} kit	Roche GmbH	11774425001
Pan B Cell Isolation Kit II	Miltenyi Biotec	130-095-813
MojoSort™ Mouse CD45 Nanobeads	BioLegend	480028
RNeasy Micro Kit	Qiagen	74004
**Chemicals**	**Company**	**Catalog Number**
Cresyl violet	Carl Roth	7651.1
Liberase	Roche	5401054001
DNase-I	Roche	11284932001
TexMACS^{TM} Medium	Miltenyi Biotech	130-097-196
Caspase 3/7 reagent	Thermo Fiser Scientific	C10427
Annexin V	BioLegend	640906
Propidium iodide	Sigma-Aldrich	P4864
Cl-amidine	Merck Millipore	506282
LDC7559	MedChemExpress	HY-111674
Dako Fluorescence Mounting Medium	Agilent Technologies	
Brain-Heart Infusion broth	Thermo Fisher Scientific	TV5090E
