# Supplementary material for: Stroke and myocardial infarction induce neutrophil extracellular trap release disrupting lymphoid organ structure and immunoglobulin secretion
Source: Nat Cardiovasc Res. 2024 Apr 23;3(5):525–40. doi: 10.1038/s44161-024-00462-8 (PMC11358010; doi:10.1038/s44161-024-00462-8)
Supplement: Supplementary file 2 — Reporting Summary [file 44161_2024_462_MOESM2_ESM.pdf]

nature portfolio

Corresponding author(s): Vikramjeet Singh
Matthias Gunzer

Last updated by author(s): 06.03.2024

Reporting Summary

Nature Portfolio wishes to improve the reproducibility of the work that we publish. This form provides structure for consistency and transparency in reporting. For further information on Nature Portfolio policies, see our Editorial Policies and the Editorial Policy Checklist.

Statistics

For all statistical analyses, confirm that the following items are present in the figure legend, table legend, main text, or Methods section.

n/a	Confirmed	
☐	☒	The exact sample size (*n*) for each experimental group/condition, given as a discrete number and unit of measurement
☐	☒	A statement on whether measurements were taken from distinct samples or whether the same sample was measured repeatedly
☐	☒	The statistical test(s) used AND whether they are one- or two-sided<br>*Only common tests should be described solely by name; describe more complex techniques in the Methods section.*
☒	☐	A description of all covariates tested
☐	☒	A description of any assumptions or corrections, such as tests of normality and adjustment for multiple comparisons
☐	☒	A full description of the statistical parameters including central tendency (e.g. means) or other basic estimates (e.g. regression coefficient) AND variation (e.g. standard deviation) or associated estimates of uncertainty (e.g. confidence intervals)
☐	☒	For null hypothesis testing, the test statistic (e.g. *F*, *t*, *r*) with confidence intervals, effect sizes, degrees of freedom and *P* value noted<br>*Give P values as exact values whenever suitable.*
☒	☐	For Bayesian analysis, information on the choice of priors and Markov chain Monte Carlo settings
☒	☐	For hierarchical and complex designs, identification of the appropriate level for tests and full reporting of outcomes
☐	☒	Estimates of effect sizes (e.g. Cohen's *d*, Pearson's *r*), indicating how they were calculated

Our web collection on statistics for biologists contains articles on many of the points above.

Software and code

Policy information about availability of computer code

Data collection	UltraMicroscope BLAZE
Data analysis	https://github.com/MMV-Lab/peyers_patch<br>https://zenodo.org/record/6302990#<br>ImageJ software<br>IMARIS<br>Trimmomatic (version 0.39)<br>Kallisto (version 0.48.0-1)<br>R (version 4.1.2)<br>Proteome Discoverer v.2.5.0.400

For manuscripts utilizing custom algorithms or software that are central to the research but not yet described in published literature, software must be made available to editors and reviewers. We strongly encourage code deposition in a community repository (e.g. GitHub). See the Nature Portfolio guidelines for submitting code & software for further information.

Data

Policy information about availability of data

All manuscripts must include a data availability statement. This statement should provide the following information, where applicable:

- Accession codes, unique identifiers, or web links for publicly available datasets
- A description of any restrictions on data availability
- For clinical datasets or third party data, please ensure that the statement adheres to our policy

All data supporting the findings in this study are available within the paper and its supplementary information. RNA sequencing, neutrophil proteomics and B cell proteomics data are provided in data source files. All RNA-seq and mass spectrometry data can be found under accession numbers GSE254410 and PXD044644 respectively. RNA sequencing reads were aligned to GRCm38 release 102 genome assembly from ENSEMBL. All details for model training and inference can be found at https://github.com/MMV-Lab/peyers_patch. The trained models are available at https://zenodo.org/record/6302990#. YhyocqvMI2x for reproducibility. Any animal materials for researchers are available upon a reasonable request to the corresponding authors.

Human research participants

Policy information about studies involving human research participants and Sex and Gender in Research.

Reporting on sex and gender	Participants for the stroke and myocardial infarction cohort were recruited without sex difference considerations and both cohorts include mixed sex patients. Respective patient characteristics can be found in materials and methods "Clinical Patient Populations" section. Control groups were selected in the same sex ratios as in the patient cohort.
Population characteristics	Humans enrolled were ischemic stroke or myocardial ischemia patients within ten days of symptom onset and with an age of 40-85. Same age-sex matched healthy controls were included without corresponding diseases.
Recruitment	Patient recruitment was done in respective clinical inpatient clinics according to NIHSS scoring, ST-elevation myocardial infarction diagnosis and informed patient consent.<br>Healthy controls were recruited via newsletter and inpatient clinic (for mild pain clinic without any other accompanying disorders) announcements. No compensation was offered. The experimentalist was blinded to the sample groups and no Bias was expected in final quantitative immunoassay based data.
Ethics oversight	The ethical approval for the use of healthy and stroke patients' plasma was granted as per the institutional ethics board committee of the University Hospital Essen (Study number: 18-8408-BO and 23-11200-BO).<br>Human plasma samples from acute ischemic stroke patients treated with or without IV DNase were collected from the NeutroStroke and IMPRESS studies at (Clinical Trial: NCT02900833 and NCT04663399)31 at the Rothschild Foundation Hospital, Paris, France with the stated approval numbers.<br>The ethical approval for the use of healthy controls and myocardial infarction patients' plasma was granted as per the institutional ethics board committee of the University Hospital Essen (Study number: 23-11200-BO) and from the Universität Münster, Münster, Germany (Study Number: 2021-424-f-S and 2021-532-f-S).

Note that full information on the approval of the study protocol must also be provided in the manuscript.

Field-specific reporting

Please select the one below that is the best fit for your research. If you are not sure, read the appropriate sections before making your selection.

☒ Life sciences ☐ Behavioural & social sciences ☐ Ecological, evolutionary & environmental sciences

For a reference copy of the document with all sections, see nature.com/documents/nr-reporting-summary-flat.pdf

Life sciences study design

All studies must disclose on these points even when the disclosure is negative.

Sample size	Each experimental group contains more than three animals per group. Respective animal numbers per experiment are stated in the figure legends. The sample size was calculated using G*Power Software. For this, F-test (ANOVA: Fixed effects, omnibus, one-way) and a priori power analysis with the given parameters (Effect size, α error and power) was performed. Number of human samples are stated in the extended data table.
Data exclusions	Laser doppler flow (LDF) was measured for the ischemic stroke surgeries and less than 80% blood flow reduction compared to baseline was considered as insufficient ischemia induction and the animals/data resulting from any related experiments were excluded from analysis.
Replication	All experiments were repeated three to five times and successfully reproduced the gained results.
Randomization	Animals were randomized before treatments and surgical procedures. Stroke patients were selected based on the diagnosis of ischemic

Randomization	stroke and stroke severity was defined according to the National Institutes of Health Stroke Scale (NIHSS). Blood samples from myocardial infarction patients were taken within four days following ST-segment elevation.
Blinding	Experimentators were blinded to the treatment groups until final data was analyzed.

Reporting for specific materials, systems and methods

We require information from authors about some types of materials, experimental systems and methods used in many studies. Here, indicate whether each material, system or method listed is relevant to your study. If you are not sure if a list item applies to your research, read the appropriate section before selecting a response.

Materials & experimental systems

n/a	Involved in the study	
☐	☒	Antibodies
☒	☐	Eukaryotic cell lines
☒	☐	Palaeontology and archaeology
☐	☒	Animals and other organisms
☐	☒	Clinical data
☒	☐	Dual use research of concern

Methods

n/a	Involved in the study	
☒	☐	ChIP-seq
☐	☒	Flow cytometry
☒	☐	MRI-based neuroimaging

Antibodies

Antibodies used

Flow cytometry:
CD45 (30-F11), Biolegend, Cat. No:103140, 1:400
CD3 (17A2),Biolegend, Cat. No:100204, 1.200
CD19 (1D3), Biolegend, Cat. No:152410, 1:500
Ly6G (1A/8), Biolegend, Cat. No:127608, 1:500
IgA (mA-6E1), Biolegend, Cat. No:127608, 1:500
CD138 (281-2), Biolegend, Cat. No:142506, 1:500
IgD (11-26C), Invitrogen, Cat. No:48-5993-82, 1:200
GL7 (GL7), Biolegend, Cat No:144604, 1:100
B220(RA3-6B2), Biolegend, Cat. No:103244, 1:250
IgG (Poly4060), Biolegend, Cat. No: 406001, 1:200
CD11b (M1/70), Invitrogen, Cat. No:48-0112-82, 1:200
IgM (II/41), eBioscience, Cat. No:17-5790-82, 1:400

In vivo depletion:
Anti-Ly6G antibody (Cat. BE0075-25,175 Bioxcell) 100 µg/mouse, anti-rat antibody (Cat. BE0122, Bioxcell) 100 µg/mouse, rat IgG2a isotype control (Cat. BE0089, Bioxcell), 100 µg/mouse and anti-CD20 antibody (Cat. BE0356, Bioxcell) 100 µg/mouse

Histological immunoflorescence /Light Sheet Fluorescence Microscopy:
CD19 (6D5), Biolegend, Cat. No:115552, 1:100
CD3 (17A2), Biolegend, Cat. No:100209, 1:100
CD31 (MEC13.3), Biolegend, Cat. No:102528, 10 µg
Gp1b-beta (anti - GPIbbeta derivative), Emfret, Cat. No:X-649, 3 µg
Ly6G (1A8), Biolegend, Cat No:127626, 1:200
citH3 (polyclonal), Abcam, ab5103, 1:200
MPO (polyclonal), Abcam, Cat No: ab9535, 1:100
EpCAM (G8.8), Biolegend, Cat. No:118222, 1:100
GL7 (GL7), Biolegend, Cat No:144606, 1:100
Donkey, anti-rabbit, Invitrogen, Cat. No:A32790, 1:200
DAPI, Carl Roth, Cat. No: 2871890-3, 1:500
CD16/32, Biolegend, Cat. No:101320, 1:1000

ELISA:
citH3 (polyclonal), Abcam, ab5103, 5 µg/ml
NE (polyclonal), Abcam, ab68672, 5 µg/ml

Validation

All antibodies were bought from commercial suppliers described in the manuscript or reporting summary. We selected antibody clones and companies that were widely used in literature. After titration tests and single- and multicolor stainings with antibodies against target populations, the analysis were made to compare efficiency and specificity. In case different lots are used, same samples are stained with old/new lots at the same time with exact dilutions to compare variability. Respective validations supplied by the manufactureres are also available in the respective websites reached by catalog number searches.

Animals and other research organisms

Policy information about studies involving animals; ARRIVE guidelines recommended for reporting animal research, and Sex and Gender in Research

Laboratory animals	C57/BL6/J 10-12 weeks old wildtype míce for stroke experiments, C57BL/6JRJ 10-12 weeks old wildtype mice for myocardial ischemia experiments and Igh-Je Jtm1Cgn/J (JHT) 12 weeks old naive mice for imaging. Mice were housed in individually ventilated cages (IVC) with dark/night cycle (12 h /12 h) and room temperature 21-23°C with 40-60% humidity.
Wild animals	The study did not use wild animals.
Reporting on sex	Only male animals were used.
Field-collected samples	The study did not use field-collected samples.
Ethics oversight	Studies were ethically approved by local authority, Landesamt für Natur, Umwelt und Verbraucherschutz Nordrhein-Westfalen, under permission numbers G1713/18; G1719/19; G1650/17; G1757/19 and conducted in acordance with the ARRIVE guidelines.

Note that full information on the approval of the study protocol must also be provided in the manuscript.

Clinical data

Policy information about clinical studies

All manuscripts should comply with the ICMJE guidelines for publication of clinical research and a completed CONSORT checklist must be included with all submissions.

Clinical trial registration	NCT02900833 and NCT04663399
Study protocol	https://clinicaltrials.gov/study/NCT02900833<br>https://www.clinicaltrials.gov/study/NCT04663399
Data collection	*Describe the settings and locales of data collection, noting the time periods of recruitment and data collection.*
Outcomes	*Describe how you pre-defined primary and secondary outcome measures and how you assessed these measures.*

Flow Cytometry

Plots

Confirm that:

☒ The axis labels state the marker and fluorochrome used (e.g. CD4-FITC).

☒ The axis scales are clearly visible. Include numbers along axes only for bottom left plot of group (a 'group' is an analysis of identical markers).

☒ All plots are contour plots with outliers or pseudocolor plots.

☒ A numerical value for number of cells or percentage (with statistics) is provided.

Methodology

Sample preparation	Tissue dissociation and single cell preparations (detailed information supplied in Methods section)
Instrument	BD FACS Aria and MACSQuant Analyzer 16
Software	FlowJo
Cell population abundance	B cells
Gating strategy	Gating is provided in Extended Data Figures S3A, B.

☒ Tick this box to confirm that a figure exemplifying the gating strategy is provided in the Supplementary Information.
